# Gut dysbiosis-induced vitamin B6 metabolic disorder contributes to chronic stress-related abnormal behaviors in a cortisol-independent manner

**DOI:** 10.1080/19490976.2024.2447824

**Published:** 2025-01-07

**Authors:** Wenxiang Qing, Huimin Chen, Xin Ma, Jie Chen, Yuan Le, Hui Chen, Jianhua Tong, Kaiming Duan, Daqing Ma, Wen Ouyang, Jianbin Tong

**Affiliations:** a Department of Anesthesiology, Third Xiangya Hospital, Central South University, Changsha, Hunan, China; b Hunan Province Key Laboratory of Brain Homeostasis, Third Xiangya Hospital, Central South University, Changsha, China; c Department of Anesthesiology, The First People’s Hospital of Yunnan Province, Kunming, Yunnan, China; d The Affiliated Hospital of Kunming University of Science and Technology, Xishan District, Kunming, Yunnan, China; e Center for Experimental Medicine, Third Xiangya Hospital, Central South University, Changsha, China; f Hunan Provincial Key Laboratory of Phytohormones and Growth Development, Hunan Agricultural University, Changsha, China; g Division of Anaesthetics, Pain Medicine and Intensive Care, Department of Surgery and Cancer, Faculty of Medicine, Imperial College London, Chelsea and Westminster Hospital, London, UK

**Keywords:** Chronic stress, gut microbial dysbiosis, vitamin B6, restraint stress

## Abstract

Chronic stress can result in various conditions, including psychological disorders, neurodegenerative diseases, and accelerated brain aging. Gut dysbiosis potentially contributes to stress-related brain disorders in individuals with chronic stress. However, the causal relationship and key factors between gut dysbiosis and brain disorders in chronic stress remain elusive, particularly under non-sterile conditions. Here, using a repeated restraint stress (RRS) rat model, we show that sequential transplantation of the cecal contents of different RRS stages to normal rats reproduced RRS-induced core phenotypes, including abnormal behaviors, increased peripheral blood corticosterone and inflammatory cytokines, and a unique gut microbial phenotype. This core phenotypic development was effectively inhibited with probiotic supplement. The RRS-induced unique gut microbial phenotypes at the genus level were positively or negatively associated with the levels of 20 plasma metabolites, including vitamin B6 metabolites 4-pyridoxic acid and 4-pyridoxate. Vitamin B6 supplement during RRS alleviated weight loss, abnormal behaviors, peripheral inflammation, and neuroinflammation, but did not affect the peripheral corticosterone levels in chronic stressed rats. Dampening inflammatory signaling via knocking out caspase 11 or caspase 1 inhibitor abolished RRS-induced abnormal behaviors and peripheral and neuroinflammation but did not decrease peripheral corticosterone in mice. These findings show that gut dysbiosis-induced vitamin B6 metabolism disorder is a new non-hypothalamic-pituitary-adrenal axis mechanism of chronic stress-related brain disorders. Both probiotics and vitamin B6 supplement have potential to be developed as therapeutic strategies for preventing and/or treating chronic stress-related illness.

## Introduction

Chronic stress is associated with psychological diseases, neurodegenerative diseases, and brain aging, and comprises one of the health epidemics in the 21^st^ century.^1,2^ Currently, the prevention of chronic stress-related brain disorders in individuals is not optimistic.^3^ It is well known that chronic stress can persistently activate both the hypothalamic-pituitary-adrenal (HPA) axis and sympathetic nervous system (SNS), resulting in damage to people’s health. However, the complexity and physiological function of stress response signals in the body makes it challenging clinically to prevent chronic stress-induced brain disorders in individuals via targeting stress response signals.^3,4^

Accumulating evidence has shown that stress exposure significantly changes the composition and relative abundance of the gut microbiota.^5–9^ Nurturing a beneficial gut microbiome with prebiotics, probiotics, or fecal microbiota transplantation has been shown to significantly alleviate chronic stress-induced organ dysfunction, systemic inflammatory responses, and increased glucocorticoid levels in animals.^8,10–13^ Moreover, previous studies have also shown chronic stress-induced brain disorders in pathogen-free mice but not in germfree mice, although HPA axis activation was noticed in both conditions of mice.^14^ Transferring the fecal microbiota from donors experiencing chronic stress to germ-free recipient mice resulted in depression-like behavior in the recipient mice.^15,16^ While novel, the experimental results regarding sterile mice lack clinical translation value. Additionally, the effects of fecal microbiota transplantation on key effectors of chronic stress, such as proinflammatory cytokines and glucocorticoids, were not reported in those studies. The causal relationship and key factors between gut microbial dysbiosis and brain disorders in chronic stress remain unknown, particularly under non-sterile conditions.

In the present work, we investigate whether chronic stress can induce gut dysbiosis and, in turn, cause further brain disorders and underlying mechanisms in rodents. We sought to find a HPA axis-independent mechanism to prevent chronic stress-related brain disorders.

## Materials and methods

### Animals

Adult Sprague Dawley rats (2 months) and C57BL/6J mice (2 months) were purchased from Central South University. Caspase 11 knockout mice were gifted by Prof. Ben Lu of the 3^rd^ Xiangya Hospital of Central South University, Changsha city, Hunan Province, China. All animals were housed in a specific pathogen-free (SPF) facility with free access to food and water. The experiments were performed in accordance with the guidelines for experimental animal use of the Central South University, Changsha, China, and the ARRIVE guidelines. The experimental protocol (LLSC(LA)2017–057) was approved by the Ethics committee of the 3^rd^ Xiangya Hospital of Central South University.

### Repeated restraint stress

Animals, including rats and mice, were subjected to restraint stress in transparent tubes without food and water from 6:00 PM to 9:00 AM daily for 4 weeks.^17^ Unstressed controls were only subjected to handling for injections, cage changes, and behavioral tests without any other restraint procedures (e.g., deprivation of water and food).

### Gut microbiota transplantation

**Figure 1.**
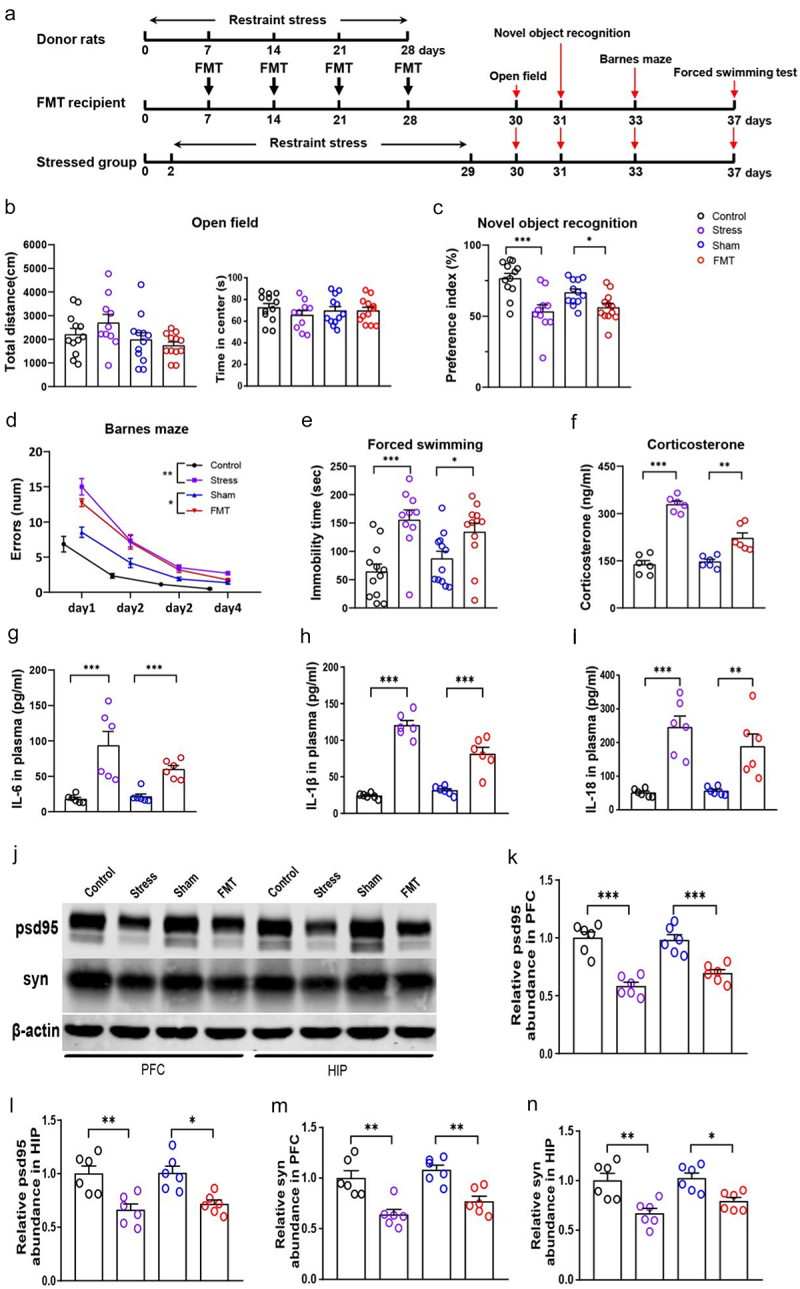
Sequential transplantation of rat cecal contents of different chronic stress stages to normal rats reproduced chronic stress-induced abnormal behaviors, increased blood corticosterone levels, and inflammatory cytokines.

In order to avoid the effect of gastric acid and oxygen exposure on the transferred microbiota, we modified the published gut microbiota transplantation protocol by transferring the gut microbiota directly into the cecum through anus.^18^ The procedures included: 1) donor rats, after being stressed for 1, 2, 3, or 4 weeks, were euthanized by 8% sevoflurane, and their cecal contents were quickly harvested into an airtight bottle, mixed, and diluted with an equal volume of saline. Notably, at each transplantation time point, all donor rats were euthanized rather than being raised for use in subsequent transplants. 2) a polyvinyl chloride catheter (2.67 mm diameter) coated with paraffin oil was gently inserted into the cecum of anesthetized naïve recipient rats through the anus advancing to the splenic flexure and hepatic flexure of the colon, finally reaching to the cecum; 3) the cecal content preparation (1.5 mL) was slowly injected into the cecum through the catheter. The experimental protocol and time frame for restraint stress and gut microbiota transplantation were as follows (Figure 1a):

(a) Time schedule for restraint stress, fecal microbiota transplantation (FMT), and behavioral tests. (b) Total distance and time in center in the open field test. (c) Preference index for a novel object in the novel object recognition task. (d) Error number in the Barnes maze. (e) Immobile time in the forced swimming test. (f) Peripheral blood corticosterone levels immediately after stress on day 29. (g) IL-6 (h) IL-1β, and (i) IL-18 levels immediately after stress on day 29. (j–n) Western blot assays of psd95 and syn in the PFC and hippocampus. Data are expressed as the mean ± SEM (*N* = 10–12 per group for behavior tests, 6 per group for blood detection, and 6 per group for western blot); *p < .05, **p < .01, ***p < .001; two-way ANOVA followed by Tukey’s multiple comparison test for comparison or unpaired *t* test where appropriate. Control: normal control group; Stress: chronic restraint stress group; Sham: sham gut microbiota transplantation group; FMT: fecal microbiota transplantation group.

The first day the donor rats were subjected to stress was defined as day 1. The donor rats were stressed from day 1 to day 28, and the fecal microbiota transplantation (FMT) recipient rats received gut microbiota transplantation at days 7,14,21, and 28. The stressed rats were treated for stress from day 2 to day 29. The FMT recipient rats and stressed rats started behavioral tests at day 30.

In the sham group, a polyvinyl chloride catheter (2.67 mm in diameter), coated with paraffin oil, was gently inserted into the cecum of anesthetized naive recipient rats via the anus. The catheter was advanced to the splenic flexure and hepatic flexure of the colon before finally reaching the cecum. Subsequently, 1.5 ml of saline was slowly infused into the cecum through the catheter.

### Probiotics and vitamin B6 administration

Probiotic capsules (BIFICO, Sine Pharmaceuticals, Shanghai, China) contained *Bifidobacterium longum* (≥1.0 × 10^7^ CFU/g), *Lactobacillus acidophilus* (≥1.0 × 10^7^ CFU/g) and *Enterococcus faecalis* (≥1.0 × 10^7^ CFU/g). Placebo capsules (also provided by Sine Pharmaceuticals) contained all ingredients except probiotics and were identical to the probiotic capsules in shape, size, and smell. As we reported previously,^19,20^ probiotic capsules were dissolved in drinking water at a ratio of 1 capsule per 30 ml of water. Our preliminary experiment found that approximately 30 ml of water was required for each rat per day. Therefore, we prepared 60 ml of drinking water with two capsules of probiotics for each rat per day. To ensure the viability of the probiotics, the probiotic solution was refreshed twice daily, regardless of whether it had been fully consumed.

Vitamin B6 (CAS:41468-25-1, Sigma-Aldrich) was dissolved in 0.9% saline. Stressed rats were administered vitamin B6 (10 mg per kg) or equal volume of 0.9% saline (vehicle control) once a day via i.p. injection.

### Behavioral tests

Rats and mice with or without stress challenges and/or treatments were subjected to open field, novel object recognition, Barnes maze, and forced swimming tests. All analyses were performed in a blinded manner.

### Open Field Test

Animals were placed in the center of the apparatus and their locomotor behaviors were recorded for 5 min using a digital camera. The total ambulatory distance was counted. The open field chamber was cleaned with 75% ethanol prior to the next trial in order to remove any odor cues.

### Novel object recognition test

This test comprised training and testing phases.^21^ In the training phase, two identical objects were placed equidistant from the center of the arena, and animals were allowed to freely explore the objects for 10 min. Twenty-four hours later, one of the familiar objects was replaced with a novel object, and the animals were allowed to freely explore it for 10 min. The behaviors were recorded using a digital camera. The preference index [defined as novel object investigation time/(novel object investigation time + familiar object investigation time)] was used to assess the learning and memory.

### Barnes Maze

As reported previously,^21^ animals were trained to locate the escaping hole in a Barnes maze four times/day for 4 days (3 min/trial and 15 min between each trial). The number of incorrectly investigated holes (termed error) during each trial were recorded. The platform surface was cleaned with 75% ethanol prior to the next trial in order to remove any odor cues.

#### Forced swimming test

The test involves two exposures to a cylindrical water tank where rats cannot reach the bottom or escape. The tank is made of transparent plexiglass, is 80 cm tall and 30 cm in diameter, and is filled with water (22–23°C) to a depth of 40 cm. For each rat, the water in the tank was changed. For the first exposure, the rats were placed in the water for 15 min (pretest session). Rats were again placed in the water 24 hours later for a 5-minute session (test session). Analyses were conducted on immobility periods. The rats were judged to be immobile whenever they stopped swimming and floated in the water, with their head just above water level. The behavior of the animals was recorded by a digital camera, and immobility time was counted and analyzed in a blind manner.

## Measurement of plasma corticosterone and inflammatory factors

As we reported previously,^21^ rats and mice with or without stress challenges and/or treatments were anesthetized with sevoflurane, and blood was quickly collected from the ventriculus dexter. After centrifugation, plasma was stored at − 80°C until further analysis. The levels of corticosterone and IL-6 in the plasma were detected according to the protocol of commercially available enzyme-linked immunosorbent assay (ELISA) kit (Corticosterone ELISA Kit, ab108821, Abcam, Cambridge, UK; IL-6 ELISA Kit, M6000B, R&D, Minneapolis, USA; IL-1β ELISA Kit, E-EL-M0037, Elabscience, China; IL-18 ELISA Kit, E-EL-M0730, Elabscience, China).

## Microbial 16S rRNA gene sequencing and analysis

Fresh cecal contents were collected from rats with or without stress challenges and treatments and stored at − 80°C until processed. Total microbial genomic DNA was extracted using the E.Z.N.A.® soil DNA Kit (Omega Bio-tek, Norcross, GA, U.S.) according to manufacturer’s instructions.^22^ The quality and concentration of DNA were determined by 1.0% agarose gel electrophoresis and a NanoDrop® ND-2000 spectrophotometer (Thermo Scientific Inc., USA) and kept at −80°C prior to further use. The hypervariable region V3-V4 of the bacterial 16S rRNA gene were amplified using primer pairs 338F (5’-ACTCCTACGGGAGGCAGCAG-3’) and 806 R (5’-GGACTACHVGGGTWTCTAAT-3’) and an ABI GeneAmp® 9700 PCR thermocycler (ABI, CA, USA). Sequencing of the PCR amplification products was performed on an Illumina Miseq platform at Majorbio Bio-Pharm Technology Co., Ltd. (Shanghai, China). Briefly, the 16S rRNA gene sequencing data was filtered and trimmed and further classified into operational taxonomic units (OTUs) within a 0.03 difference (equivalent to 97% similarity). A representative set of sequences was then generated and assigned taxonomy using the SILVA database (Release115, http://www.arb-silva.de). Taxonomic community structure and phylogeny were analyzed through visualization of the microbial diversities and abundances of different samples.

## Metabolomics

Blood samples were collected from the ventriculus dexter of the rat and then centrifuged at 3000 rpm for 10 min to extract the plasma for further analysis. Samples were kept at −80°C prior to further use. For extraction, plasma samples were thawed on ice. Then 100 μl of the samples were taken and placed in an EP tube, and then extracted with 300 μl methanol and 10 μl internal standard, followed by vortexing for 30 s, ultrasound treatment for 10 min (incubated in ice water), and then incubation at −20°C for 1 h to precipitate proteins. Then the samples were centrifuged at 13,000 rpm for 15 min at 4°C. The supernatant (200 μl) was transferred to liquid chromatograph-mass spectrometer (LC-MS) vials. The quality control sample was prepared by mixing an equal aliquot of the supernatant from all of the samples. LC-MS analyses were performed using a ultra-high-performance liquid chromatography (UHPLC) system (1290, Agilent Technologies) with a UPLC HSS T3 column (1.8 μm 2.1 × 100 mm, Waters) coupled to Q Exactive (Orbitrap MS, Thermo). The QE mass spectrometer was used for acquiring MS/MS spectra on an information-dependent basis (IDA) during a LC/MS experiment. Then, the acquisition software (Xcalibur 4.0.27, Thermo) continuously evaluated the full-scan survey MS data as it collected and triggered the acquisition of MS/MS spectra depending on preselected criteria. For data processing, MS raw data files were converted to the mzXML format by ProteoWizard and processed by R package XCMS (version 3.2). The process includes peak deconvolution, alignment, and integration. Minfrac and cutoff were set as 0.5 and 0.3, respectively. In-house MS2 database was applied for metabolites identification.

The LC/MS generated raw data were manipulated with the TMBQ software (version 1.0, Metabo-Profile, Shanghai, China) to carry out peak detection, normalization, and determination of metabolite quantities. SIMCA software was employed for statistical evaluations, covering methods such as PCA, OPLS-DA, univariate examination, and metabolic pathway assessment. Within the metabolomic analyses, both multivariate methods (PCA, OPLS-DA) and univariate approaches (Student’s *t* test, Mann – Whitney-Wilcoxon U test, ANOVA, and correlation analysis) were conducted. The computation approach was derived from a widely-employed statistical package in R Studio (http://cran.r-project.org/). To examine the relationships between microbial groups and metabolites, Spearman’s rank correlation analysis was performed.

## Plasma 4-pyridoxic acid quantitative detection

The method outlined for the quantification of 4-pyridoxic acid in plasma involved several steps: sample pretreatment, derivatization, chromatographic separation, and fluorescence detection. Initially, the plasma sample was mixed with trichloroacetic acid to aid in the removal of proteins. Next, under alkaline conditions with the presence of cyanide, the pyridoxal 5’-phosphate was transformed into 4-pyridoxic acid 5’-phosphate, and orthophosphoric acid was then added to acidify the resulting extract. Subsequently, reverse-phase high-performance liquid chromatography was employed to separate the modified sample, with 4-pyridoxic acid being eluted between the 8.2 to 8.7 minutes. A fluorescence detector was utilized to identify the separated 4-pyridoxic acid, with excitation and emission wavelengths set at 325 nm and 418 nm, respectively. These steps collectively enabled the method to quantitatively assess the content of 4-pyridoxic acid in plasma. Moreover, a standard solution of 4-pyridoxic acid was used to develop a calibration curve, which was critical for ensuring the accuracy of the quantification process.

## Western blot

Hippocampus tissue from rats with or without stress challenges and treatments were homogenized mechanically in RIPA buffer (Beyotime, ShangHai, China), and then the proteins were extracted. Samples containing 20–80 μg protein were separated by 10% SDS-polyacrylamide gel electrophoresis and transferred to nitrocellulose membrane (NC) membranes, which were subsequently blocked using 5% milk. Blots were detected using primary antibodies against β-actin (1:2000, Proteintech, WuHan, China), PSD95 (1:1000, Cell Signaling Technology, Massachusetts, USA), and synaptophysin (1:500, Proteintech, WuHan, China). Subsequently, blots were incubated with the fluorescent secondary antibodies.

## Immunostaining

Immunostaining was conducted on 20-μm thick coronal brain sections, selectively labeled with either Iba1 or DCX, and on 5-μm thick sections stained for alkaline phosphatase (ALP), obtained from rats that had been exposed to stress challenges and received various treatments, as well as from untreated control rats. After treating with 3% H_2_O_2_ and blocking with 5% BSA, sections of the brains were incubated in primary antibodies (rabbit anti-Iba1: 1:1000, Wako, Japan; rabbit anti-DCX: 1:500, Cell Signaling Technology, Massachusetts, USA; rabbit anti-ALP: 1:200, Proteintech, WuHan, China) at 4°C overnight. Subsequently, the sections were washed three time with PBS and then the secondary antibody (goat anti-rabbit: 1:200, Jackson ImmunoResearch, United States) was added for 2 hours at room temperature. The sections were coverslipped with Vectamount mounting medium with DAPI (Vector labs H-1000). Images were acquired using LSM800 confocal microscope and Zen 2009 image acquisition software (Carl Zeiss, Jane, Germany). The positive area in these images was counted and analyzed by a trained technician who was blinded to experimental conditions.

## Peripheral blood white cell counting

Under anesthesia, blood samples were collected from the ventriculus dexter of rats with or without stress challenges and treatments into EDTA-coated tubes. After a three-fold dilution with saline, the blood was rapidly tested by a Mindray BC-5300 blood analyzer.

## Quantitative real-time polymerase chain reaction (rt-qPCR) assay

Total RNA was extracted using the Trizol Reagent (Invitrogen, United States) and reverse transcribed into complementary DNA using a cDNA Synthesis Kit (GeneCopoeia, United States) according to the manufacturer’s instructions. Quantitative real-time polymerase chain reaction (RT-qPCR) was performed with the mRNA qPCR mix (GeneCopoeia, United States) accordingly. Primers for all assayed genes were determined using reported sequences (β-actin: F’-TCTATCCTGGCCTCACTGTC, R’- AACGCAGCTCAGTAACAGTCC, IL-6: F’- GGTCTGTTGTGGGTGGTATCC, R’- CCAGTTGCCTTCTTGGGACT).^23^ The reaction was performed using LightCycler®480II analyzer (Roche, Mannheim, Germany).

## Statistical analyses

Data were presented as a dot plot and expressed as the mean ± SEM. Data were analyzed with a Student’s *t* test, Wilcoxon rank sum test, or a one- or two-way ANOVA (two-tailed) followed by Tukey’s multiple comparison test wherever appropriate. All analyses were performed with GraphPad Prism 8 (GraphPad Software Inc., La Jolla, CA, USA). The level of significance was set at *p* < 0.05.

## Results

### Sequential transplantation of rat cecal contents from different chronic stress stages to normal rats reproduced chronic stress-induced abnormal behaviors, increased blood corticosterone levels and inflammatory cytokines, and an unique gut microbiota phenotype

To assess the role of gut dysbiosis in the development of brain disorders in individuals with chronic stress, we stressed specific pathogen-free rats following a well-established chronic restraint stress paradigm for 28 days.^17^ Cecal contents from rats stressed for 7, 14, 21, or 28 days were successively transplanted into the cecum of normal rats through the anus once a week for 4 weeks (Figure 1a). We found that rats that received four transplantations of stressed cecal contents (fecal microbiota transplantation (FMT) group) showed an impairment in novel object recognition, the Barnes maze, and forced swimming test, and increased peripheral blood corticosterone and inflammatory factors levels (IL-6, IL-1β, and IL-18), similar to the rats stressed for 28 days (Figure 1b–i). In line with the observed behavioral alterations, we noted a marked reduction in the expression of post-synaptic density protein 95 (psd95) and synapsin (syn) within the prefrontal cortex (PFC) and hippocampus (HIP) of rats in both the chronic stress and FMT groups (Figure 1j–n). This finding indicated a strong correlation between disruptions in the gut microbiota and the core phenotypes induced by chronic stress.

**Figure 2.**
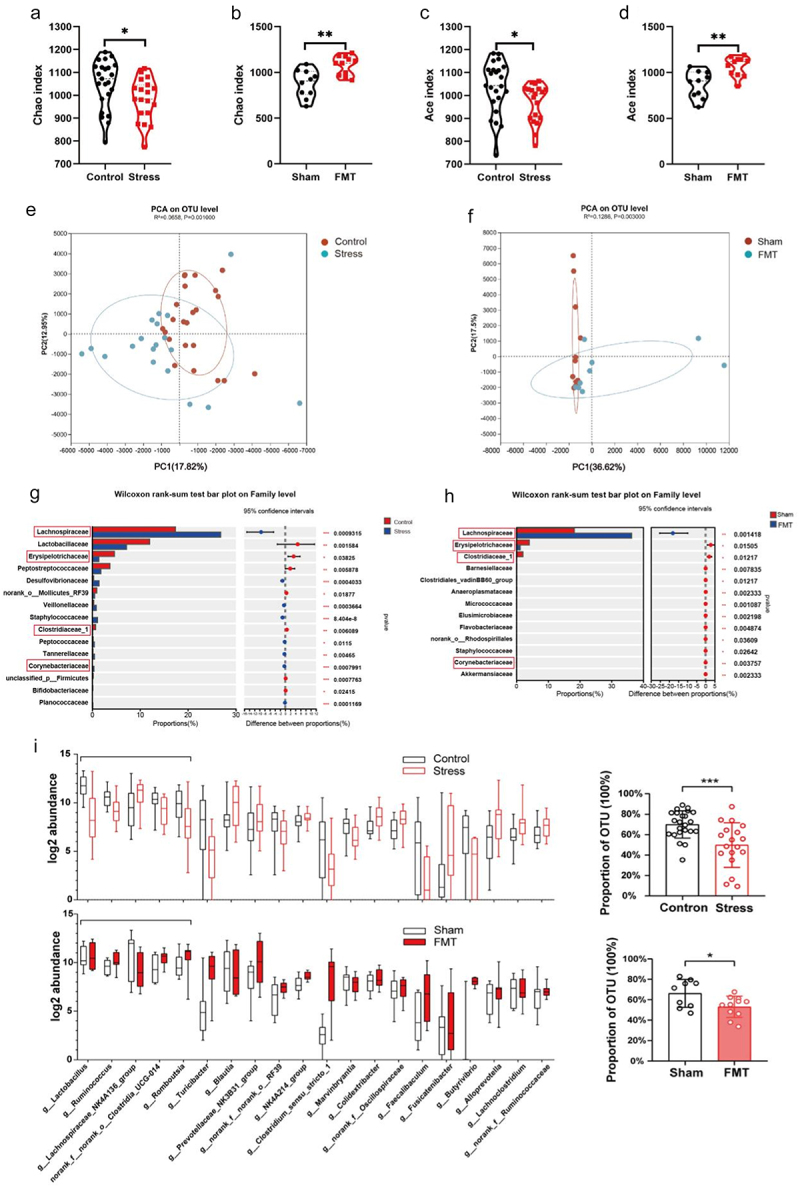
Sequential transplantation of rat cecal contents of different chronic stress stages to normal rats reproduced the chronic stress-induced gut microbiota phenotype.

To further investigate the role of the gut microbiota in stress-induced brain disorders, we conducted 16S rRNA gene sequencing on the aforementioned groups (Figure 2 and Figure S1). Alpha diversity was assessed using the Chao and Ace indices across the different groups. Investigation of the Chao diversity and Ace indices showed a notable decrease in the overall microbiota abundance in the stress group compared to the control group (Figures 2a and 2c). Compared to the Sham group, fecal microbiota transplantation significantly increased the α-diversity of the gut microbiota (Figures 2b and 2d). Moreover, principal component analysis was conducted to illustrate the scattered distribution of data points on the scatterplots of the various groups, indicating that the compositions of the stressed group were significantly different from those of the control group, and the same changes indicated that the transplanted microbiota altered the gut microbiota of the rats (Figure 2e–f). At the family level, the relative abundance of Lachnospiraceae, Erysipelotrichaceae, Clostridiaceae, and Corynebacteriaceae represented a common set of differentially abundant microbiota between the Control vs. Stress groups and the Sham vs. FMT groups (Figure 2g–h). Further analysis at the genus level revealed that 20 gut microorganisms with an OTU count higher than 100 exhibited inter-group differences between the normal control group and the stressed rats (Figure 2i). Among those, the percentage of the top 5 gut microorganisms in the stressed rats was significantly less than that of the normal controls (Figure 2i). A similar percentage change in the top 5 gut microorganisms was also detected between rats with four transplantations of stressed cecal contents or the sham-treated controls (Figure 2i). These data revealed that a stressed gut microbiota promotes the development of chronic stress-induced core phenotypes, including abnormal behaviors and increased corticosterone levels and inflammatory cytokines in peripheral blood.

(a) Microbial community α-diversity (measured by Chao index) of Control vs. Stress groups. (b) Microbial community α-diversity (measured by Chao index) of Sham vs. FMT groups. (c) Microbial community α-diversity (measured by Ace index) of Control vs. Stress groups. (d) Microbial community α-diversity (measured by Ace index) of Sham vs. FMT groups. (e) Principal component analysis (PCA) of Control vs. Stress groups. (f) Principal component analysis (PCA) of Sham vs. FMT groups. (g) The intestinal microbiota was significantly different at the genus level between the Control vs. Stress groups. (h) The intestinal microbiota was significantly different at the genus level between the Sham vs. FMT groups. (i) The percentage of the top 5 gut microorganisms out of 20 gut microorganisms with OTUs higher than 100 and the significant inter-group difference. Cecal microbiota samples were collected immediately after stress on day 29, sequenced via 16S rRNA gene sequencing, and analyzed. Control: normal control group; Stress: chronic restraint stress group; Sham: sham gut microbiota transplantation group; FMT: fecal microbiota transplantation group.

### Nurturing a beneficial gut microbiota with probiotics prevented the development of both abnormal behaviors and increased blood corticosterone and inflammatory cytokines levels in chronically stressed rats

**Figure 3.**
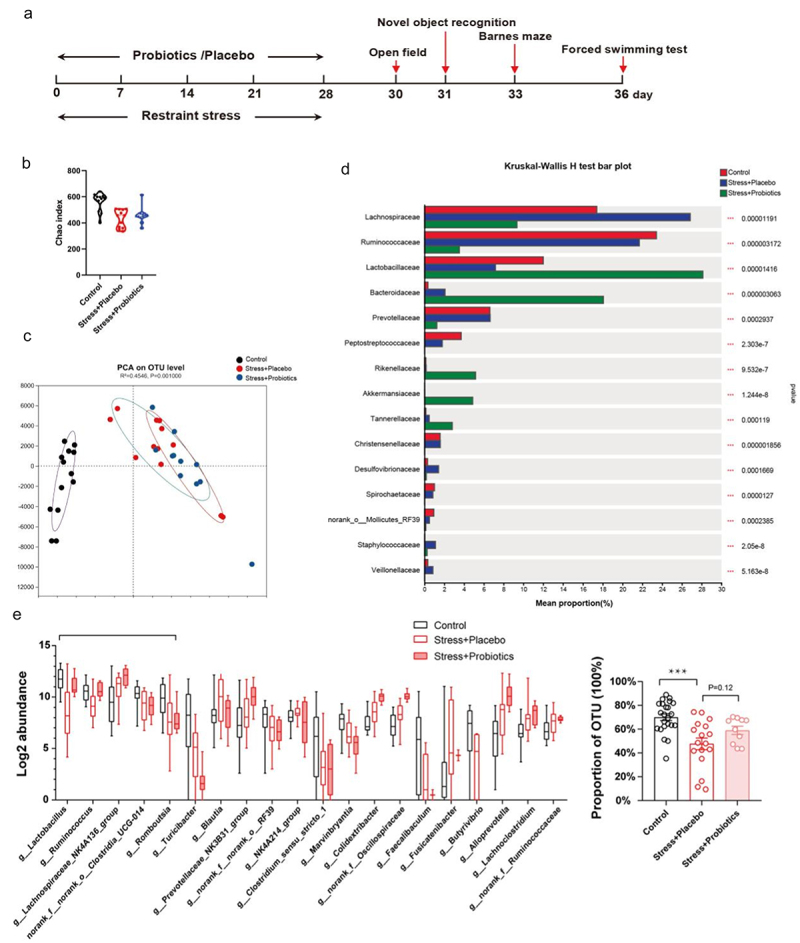
Probiotic supplementation during chronic stress partly prevented stress-induced gut dysbiosis.

**Figure 4.**
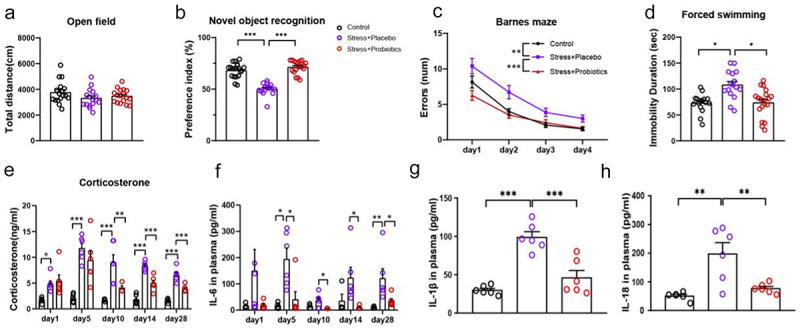
Nurturing a beneficial gut microbiota with probiotics prevented the development of abnormal behaviors, increased blood corticosterone, and inflammatory cytokines in chronically stressed rats.

The above FMT experiments showed an important role of gut dysbiosis in the development of chronic stress-induced core phenotypes. To confirm the role of gut dysbiosis, we next tested whether prevention of chronic stress-induced gut dysbiosis can prevent the development of core phenotypes. Probiotics are commonly used to nurture a beneficial gut microbiome and have been shown to have therapeutic effects on the cognitive performance of moderately stressed healthy adults.^24^ Thus, probiotic supplementation was used as a method to prevent gut dysbiosis during chronic stress (Figure 3a). We found that probiotic supplementation during chronic stress partly normalized chronic stress-induced gut dysbiosis (Figure 3b-d, Figure S2). Compared to the control group, the stress group exhibited a notable reduction in the levels of Lactobacillaceae and Lactobacillus, whereas supplementation with probiotics led to a significant increase in these bacteria (the primary components of the probiotic) (Figure 3d and Figure S2). Benefiting from the improvement in gut ecosystem, there were also changes in some other microbiota in the gut. Interestingly, there was a significant change in Lachnospiraceae in the microbiota transplantation experiment, which showed similar changes after probiotic supplementation (Figure 3d and Figure S2). More importantly, the top 5 gut microbiota in control rats exhibited a notable decrease in the stress group, which was partially restored through probiotic supplementation (Figure 3e). Next, we investigated the effects of probiotic supplementation on the core phenotypes induced by stress and found that probiotic supplementation significantly improved the abnormal behaviors of stressed rats in the novel object recognition, Barnes maze, and forced swimming tests, as well as reduced the levels of peripheral blood corticosterone and IL-6 levels (Figure 4a–h).

(a) Time schedule for restraint stress, probiotic supplementation, and the behavioral tests. (b) Microbial community α-diversity (measured by Chao index). (c) Principal component analysis (PCA) at the OTU level. (d) Significantly different intestinal microbiota at the genus level. (e) The percentage of the top 5 gut microorganisms out of 20 gut microorganisms with OTUs higher than 100, and the significant inter-group difference. Cecal microbiota samples were collected immediately after stress on day 29, sequenced via 16S rRNA sequencing, and then analyzed. Control: normal control group; Stress + Placebo: rats were administered placebo capsules via drinking water once a day throughout the restraint stress period; Stress + Probiotics: rats were administered probiotic capsules via drinking water once a day throughout the restraint stress period.

(a) Total distance in open field test. (b) Preference index for a novel object in novel object recognition task. (c) Error number in the Barnes maze. (d) Immobile time in the forced swimming test. (e) Peripheral blood corticosterone levels were measured in the morning of the days 1, 5, 10, 14, and 28 of restraint stress. (f) IL-6 (g) IL-1β, and (h) IL-18 levels immediately after stress on day 29. Data are expressed as mean ± the SEM (*N* = 11–25 per group for behavior tests, 4–6 per group for blood detection); * *p* < .05, ** *p* < .01, *** *p* < .001; one- or two-way ANOVA followed by Tukey’s multiple comparison test for comparison. Control: normal control group; Stress + Placebo: rats were administered placebo capsules via drinking water once a day throughout the restraint stress period; Stress + Probiotics: rats were administered probiotic capsules via drinking water once a day throughout the restraint stress period.

### Chronic stress-induced gut dysbiosis distorted plasma metabolites including vitamin B6 metabolism

**Figure 5.**
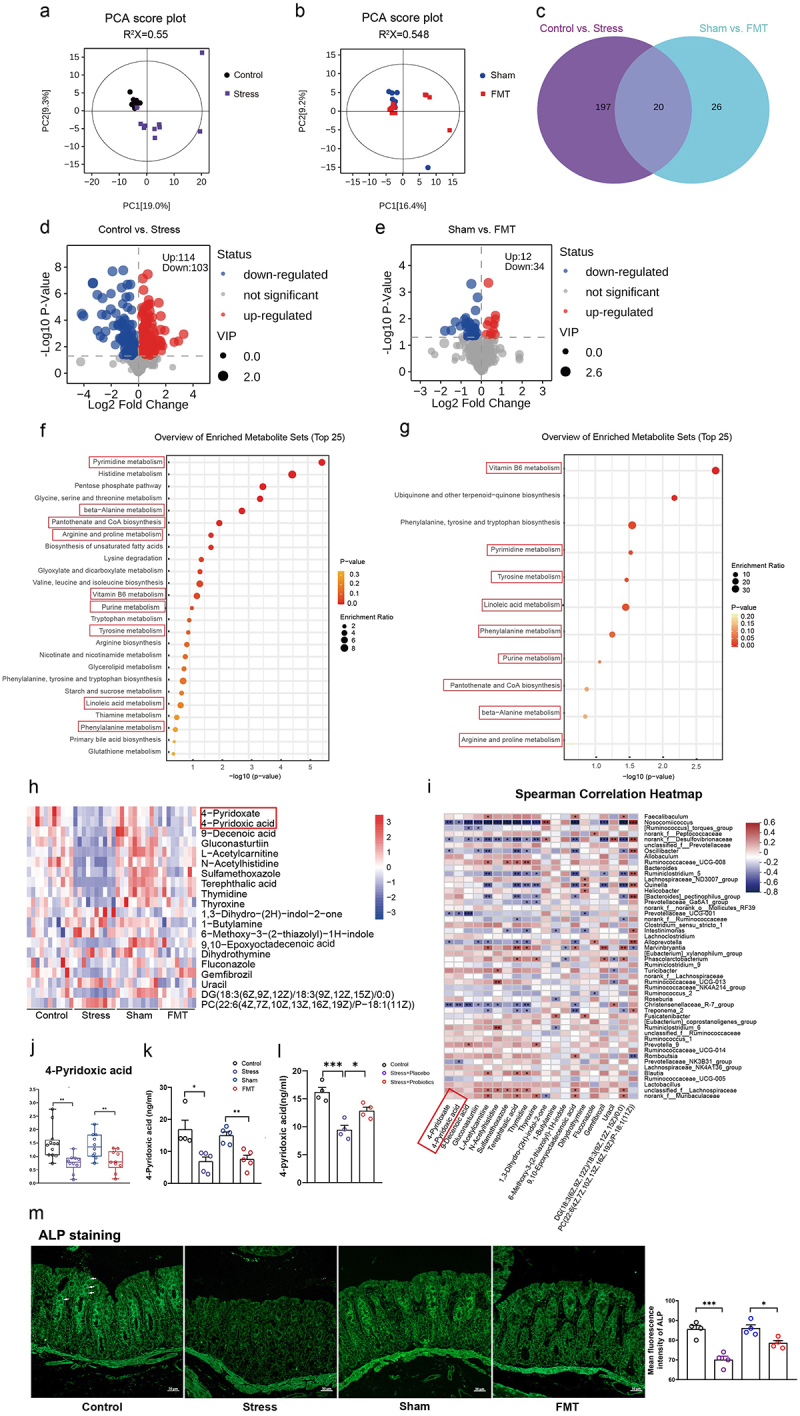
Chronic stress-induced gut dysbiosis altered plasma metabolites, including vitamin B6 metabolism.

The two experiments described above established the causal relationship between gut dysbiosis and abnormal behavioral changes in individuals with chronic stress. We next explored the key players for such changes by comparing the differences in plasma metabolites in rats with chronic stress, stressed cecal content transplantation, sham transplantation, and no treatment. PCA and OPLS-DA analyses revealed significant differences between the stress group and the control group; furthermore, compared to the sham group, fecal microbiota transplantation also led to notable changes in metabolites (Figure 5a-b and Figure S3A – S3B). We found 217 differentially expressed metabolites between the control and stress groups, while only 46 metabolites demonstrated significant variances between the sham and transplantation groups (Figure 5c, Figure S3C – S3D). The volcano plot illustrates the differential expression of metabolites among the groups. Compared to the control group, the stress group had 114 upregulated and 103 downregulated metabolites, while the transplantation group had 12 upregulated and 34 downregulated metabolites compared to the sham group (Figure 5d–e). Next, we conducted KEGG signaling pathway analysis using differentially expressed metabolites. Notably, nine of the signaling pathways affected by the changes in metabolites resulting from fecal microbiota transplantation were consistent with those associated with stress-induced changes in metabolites (Figure 5f–g). The nine shared metabolic pathways between the Control vs. Stress and Sham vs. FMT comparisons included vitamin B6 metabolism, purine metabolism, and other related pathways (Figure 5f–g). A total of 20 differentially expressed metabolites were found to be shared between the Control vs. Stress and Sham vs. FMT comparisons (Figure 5h). Among the 20 differential plasma metabolites identified, we found two members of the vitamin B6 family, 4-pyridoxic acid and 4-pyridoxate – both known for their anti-inflammatory, anti-stress, and anti-oxidative properties.^25–28^ Therefore, we presumed that vitamin B6 may be a mediator of gut dysbiosis-related core phenotypes in chronically stressed rats. The decline of 4-pyridoxic acid levels in both chronical stressed rats and those that underwent fecal microbiota transplantation (FMT) was corroborated by quantitative mass spectrometry (Figure 5j–k). Moreover, the supplementation with probiotics effectively restored the reduced concentrations of 4-pyridoxic acid in the stressed rats (Figure 5l). Intestinal epithelial alkaline phosphatase (ALP) is a pivotal enzyme involved in the absorption of vitamin B6 into the bloodstream, enabling the entry of vitamin B6 into the body and the subsequent execution of its biological functions.^29^ The expression of ALP in intestine epithelial cells was significantly decreased in rats with chronic stress or stressed cecal contents transplantation (Figure 5m). To explore the potential relationship between the diversity and abundance of the gut microbiota and the levels of plasma metabolites, we conducted a Spearman’s correlation analysis and generated a heatmap (Figure 5i). The results indicated that the majority of the altered gut microbiota at the genus level exhibited positive or negative associations with the levels of the aforementioned 20 distinct plasma metabolites, and 4-pyridoxic acid was positively correlated with prevotella and negatively correlated with prevotellaceae_NK3B31_group, Christensenellaceae_R-7_group, prevotellaceae_UCG-001, Nosocomiicoccus. Additionally, 4-pyridoxate was negatively correlated with Christensenellaceae_R-7_group, alloprevotella,prevotellaceae_UCG-001, oscillibacter, desulfovibrionaceae, nosocomiicoccus (Figure 5i). These findings emphasized the role of the gut microbiota in modulating plasma metabolites, which suggests that chronic stress-induced alterations in the gut microbiota can lead to changes in plasma metabolites, particularly those involved in vitamin B6 metabolism.

(a) Principal component analysis (PCA) of the Control vs. Stress groups. (b) Principal component analysis (PCA) of the Sham vs. FMT groups. (c) Venn diagrams reflecting the overlap of various metabolites. (d) Volcano plot analysis of differential metabolites between the Control and Stress groups. (e) Volcano plot analysis of differential metabolites between the Sham and FMT groups. (f) KEGG pathway enrichment analysis of the metabolite profiles of the Control vs. Stress groups. (g) KEGG pathway enrichment analysis of the metabolite profiles of the Sham vs. FMT groups. (h) A heatmap displaying the 20 shared differentially abundant metabolites between the Control vs. Stress and Sham vs. FMT groups. (i) Heatmap of Spearman correlation analysis between the gut microbiota and metabolites. (j) The relative level of 4-pyridoxic acid in the plasma of rats immediately after stress on day 29 in untargeted metabolomics. (k) Targeted validation of the relative level of 4-pyridoxic acid in plasma of the FMT rat group immediately after stress day 29. (l)Targeted validation of the relative level of 4-pyridoxic acid in the plasma of probiotics intervention rat group immediately after stress day 29. (m) Representative micrographs of alkaline phosphatase (ALP) staining in the intestine (white arrow). Scale bar = 50 µm Control: normal control group; Stress: chronic restraint stress group; Sham: sham gut microbiota transplantation group; FMT: fecal microbiota transplantation group.

### Intraperitoneal injection of vitamin B6 during stress alleviated weight loss, abnormal behaviors, peripheral inflammation and neuroinflammation, but did not affect peripheral corticosterone level in chronically stressed rats

**Figure 6.**
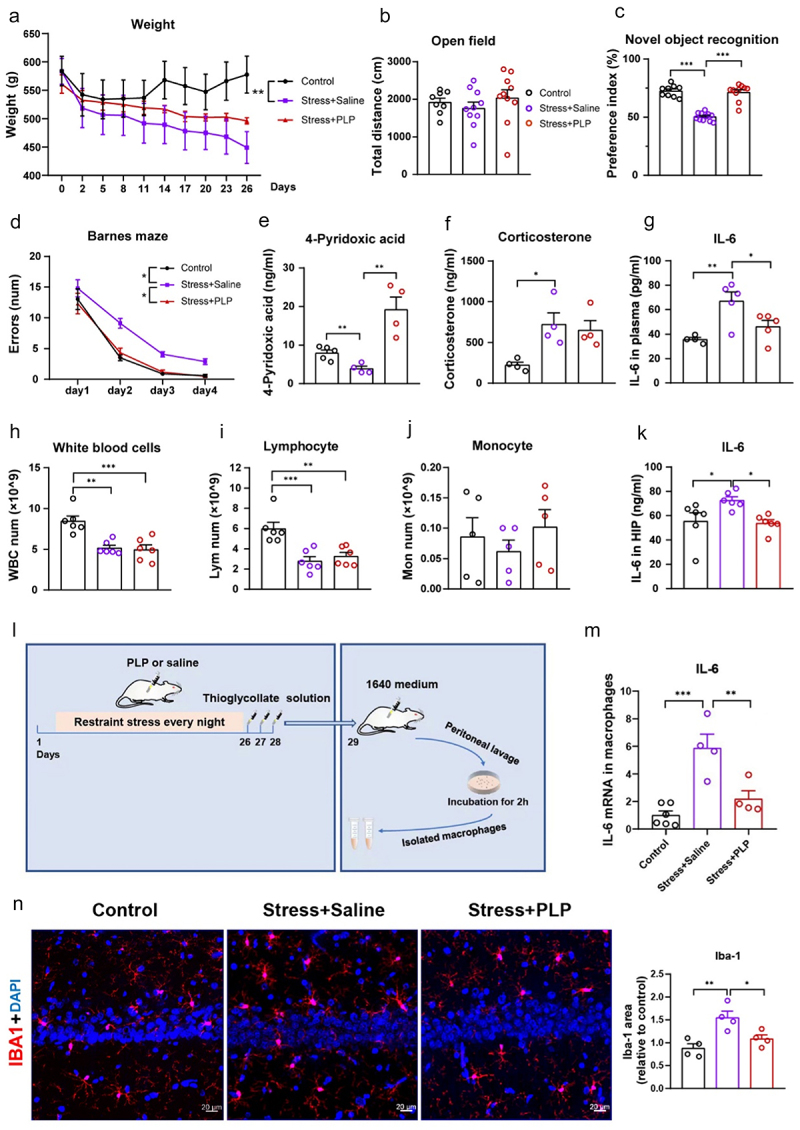
Intraperitoneal injection of vitamin B6 during stress alleviated weight loss, abnormal behaviors, peripheral inflammation and neuroinflammation, but did not affect peripheral corticosterone level in chronic stressed rats.

To further investigate the key role of vitamin B6 in gut dysbiosis-induced core phenotypes of chronically stressed rats, vitamin B6 was injected intraperitoneally into rats during chronic stress. Vitamin B6 supplement during chronic stress limited the weight loss of stressed rats, normalized abnormal behaviors assessed with novel object recognition and the Barnes maze test, and decreased blood IL-6, but did not affect the blood corticosterone level and peripheral blood white cells including lymphocyte and monocyte counts (Figure 6a–j). In addition, the vitamin B6 supplement inhibited neuroinflammation and neurogenesis impairment (Figures 6k, n, Figure S4A), and reversed the stress induced reduction of postsynaptic density protein 95 (psd95) and synapsin (syn) protein levels in the hippocampus and prefrontal cortex (Figure S4B-S4C). It also significantly decreased IL-6 gene expression in intraperitoneal macrophages from stressed rats (Figure 6l–m). These data showed that vitamin B6 deficits mediate gut dysbiosis-related core phenotypes of chronically stressed rats, and vitamin B6 supplementation inhibited chronic stress-induced systemic and neural inflammation without affecting the peripheral corticosterone.

(a) Weight changes during chronic stress. (b) Total distance in the open field test on the 1^st^ day after 28 days of restraint stress. (c) Preference index for novel object in novel object recognition task on the 3^rd^ day after 28 days of restraint stress. (d) Error number in the Barnes maze from days 4–8 after 28 days of restraint stress. (e) Concentrations of 4-pyridoxic acid in the plasma of rats immediately after stress on day 29. (f) Peripheral blood corticosterone levels immediately after stress on day 29. (g) Peripheral blood IL-6 levels immediately after stress on day 29. (h) Peripheral white blood cell numbers immediately after stress on day 29. (i) Peripheral lymphocyte numbers immediately after stress on day 29. (j) Peripheral monocyte numbers immediately after stress on the day 29. (k) Hippocampal IL-6 levels immediately after stress on day 29. (l) Experimental time course for restraint stress, the administration of PLP, saline, and thioglycolate solution, peritoneal macrophage collection, peritoneal macrophage purification, and IL-6 mRNA detection. (m) IL-6 mRNA quantification in peritoneal macrophages. (n) Representative images (left panel) and quantification (right panel) of Microglial activation (IBA1, red) in hippocampus of rats (bar = 20µm). Data are expressed as the mean±SEM (N = 8–11 per group for behavior tests, 4–6 per group for blood detection, and 3–6 per group for PFC or HIP detection); *p<0.05, **p<0.01, ***p<0.001; one- or two-way ANOVA followed by Tukey’s multiple comparison test for comparison or unpaired *t* test where appropriate. Control: normal control group; Stress+Saline: rats was injected intraperitoneally with saline once a day throughout the restraint stress period; Stress+PLP: rats were injected intraperitoneally with PLP once a day throughout the restraint stress period.

### Inflammation acting as a downstream effector of corticosterone plays a key role in the pathogenesis of chronic stress-induced abnormal behaviors

**Figure 7.**
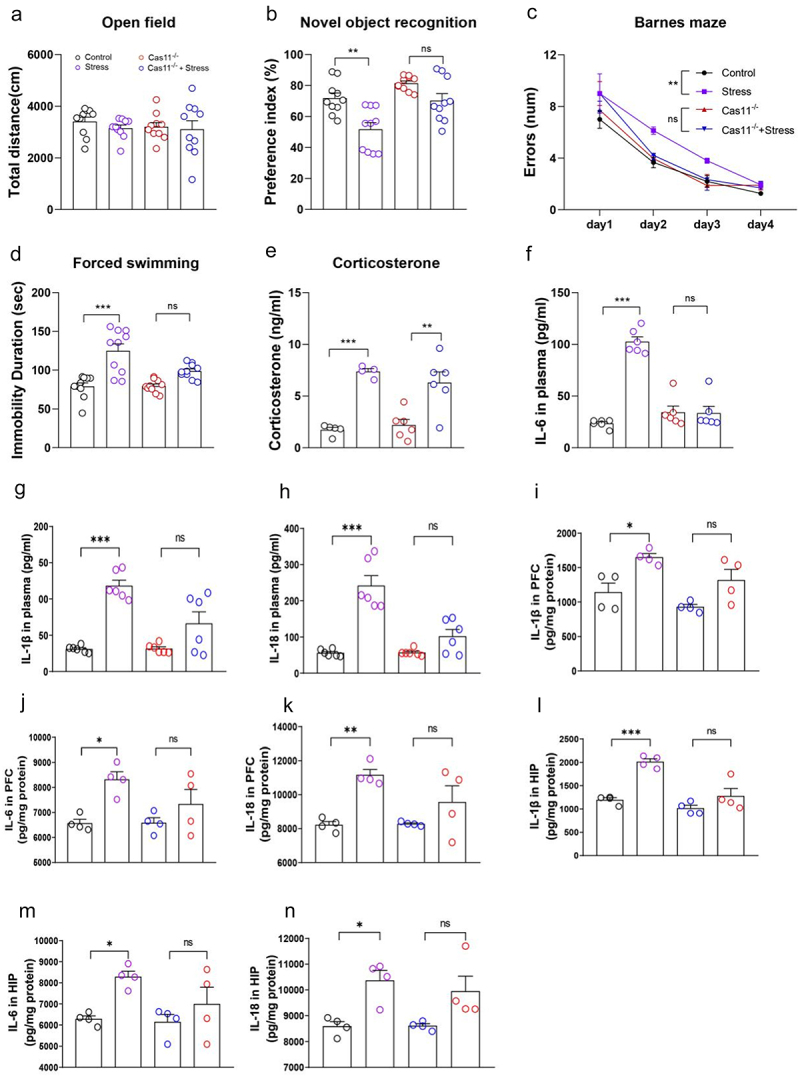
Inflammation acting as a downstream effector of corticosterone plays a key role in the pathogenesis of chronic stress-induced abnormal behaviors in caspase 11 knockout mice.

**Figure 8.**
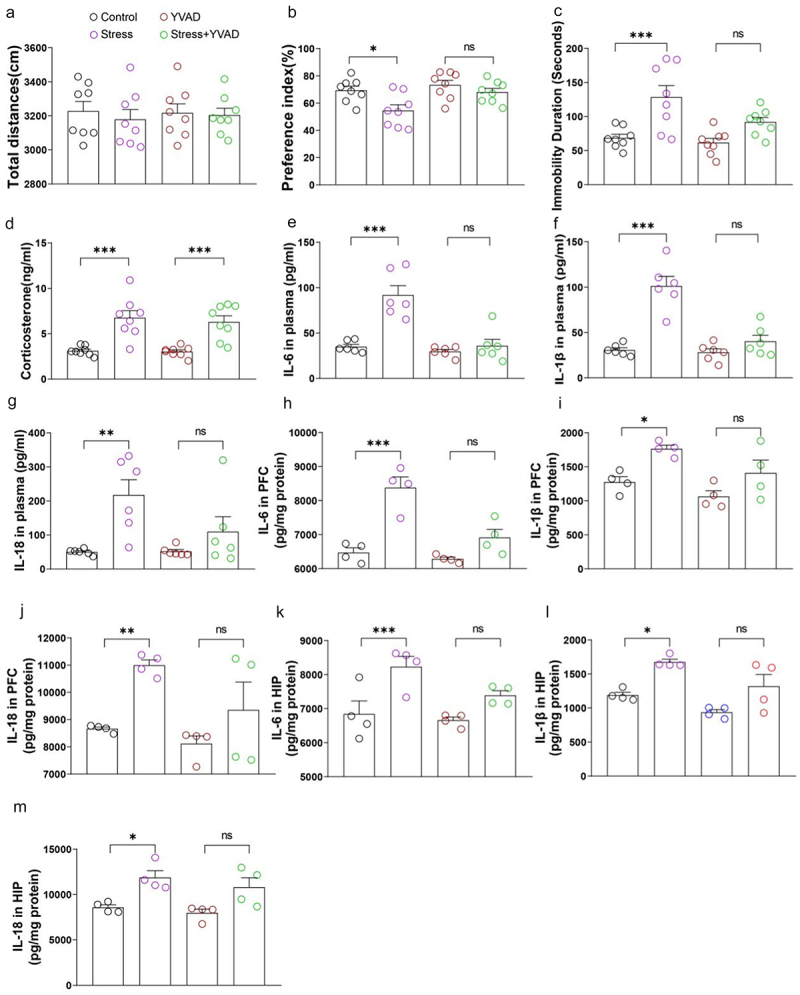
Inflammation acting as a downstream effector of corticosterone plays a key role in the pathogenesis of chronic stress-induced abnormal behaviors in mice pretreated with caspase 1 inhibitors.

Rodents with chronic stress are characterized as having high levels of peripheral blood corticosterone and inflammatory cytokines as reported above. However, Vitamin B6 supplement inhibited inflammatory cytokine release but did not affect peripheral blood corticosterone in stressed rats (Figure 6f–g). Thus, we evaluated the relationship between increased corticosterone and inflammatory cytokines in the development of chronic stress-induced abnormal behaviors. Caspase 11 and caspase 1 are members of the inflammatory caspase family and serve as important mediators of innate immunity.^30^ In wild type mice, chronic stress induced abnormal behaviors assessed with novel object recognition, Barnes maze test and Forced swimming test, and increased the levels of blood corticosterone and peripheral and neuroinflammation (Figure 7). In contrast, in Caspase 11 knockout mice or mice pretreated with caspase 1 inhibitors (Ac-YVAD-cmk), chronic stress did not induce abnormal behaviors or the increase of peripheral and neuroinflammation; instead, it elevated the level of corticosterone in the peripheral blood (Figures 7 and 8). These results suggest that stress induced inflammation is likely a downstream effector of corticosterone, and Vitamin B6 may only work below corticosterone level.

(a) Total distance in open field on the 1st day after 28 days of restraint stress. (b) Preference index for novel object in the novel object recognition task on the 3rd day after 28 days of restraint stress. (c) Error number in the Barnes maze from days 4–8 after 28 days restraint stress. (d) Immobile time in the forced swimming test on the 9th day after 28 days of restraint stress. (e) Peripheral blood corticosterone levels immediately after stress on day 29. (f) IL-6, (g) IL-1β, and (h) IL-18 levels in peripheral blood immediately after stress on the day 29. (i) IL-6, (j) IL-1β, and (k) IL-18 levels in PFC immediately after stress on the day 29. (l) IL-6, (m) IL-1β, and (n) IL-18 levels in the hippocampus immediately after stress on the day 29. Data are expressed as the mean ± SEM (*N* = 10–11 per group for behavior tests and 4–6 per group for blood measurements); *p < 0.05, **p < 0.01, ***p < 0.001; two-way ANOVA followed by Tukey’s multiple comparison test for comparison or unpaired *t* test where appropriate. Control: normal control group of wild-type mice; stress: chronic restraint stress group of wild-type mice; Cas11^−/−^: normal control group of caspase 11 knockout mice; Cas11^−/−^ + Stress: chronic restraint stress group of caspase 11 knockout mice.

(a) Total distance in open field on the 1st day after 28 days of restraint stress. (b) Preference index for novel object in the novel object recognition task on the 3rd day after 28 days of restraint stress. (c) Immobile time in the forced swimming test on the 9th day after 28 days of restraint stress. (d) Peripheral blood corticosterone levels immediately after stress on day 29. (e) IL-6, (f) IL-1β, and (g) IL-18 levels in peripheral blood immediately after stress on the day 29. (h) IL-6, (i) IL-1β, and (j) IL-18 levels in PFC immediately after stress on the day 29. (k) IL-6, (l) IL-1β, and (m) IL-18 levels in the hippocampus immediately after stress on the day 29. Data are expressed as the mean ± SEM (*N* = 10–11 per group for behavior tests, 4–6 per group for blood measurements and 4 per group for PFC or HIP detection); *p < 0.05, **p < 0.01, ***p < 0.001; two-way ANOVA followed by Tukey’s multiple comparison test for comparison or unpaired *t* test where appropriate. Control: normal control group of wild-type mice; stress: chronic restraint stress group of wild-type mice; YVAD: normal control group treated with Ac-YVAD-cmk once a day; Stress + YVAD: Mice received an intraperitoneal injection of Ac-YVAD-cmk prior to daily stress exposure.

## Discussion

In this study, we found that sequential transplantation of cecal contents from rats undergoing different stages of chronic stress to normal rats reproduced chronic stress-induced core phenotypes, including abnormal behaviors, increased peripheral blood corticosterone and inflammatory cytokine levels, and a unique gut microbial phenotype. This core phenotypic development was effectively inhibited with probiotic supplement. The unique gut microbial phenotypes induced by chronic stress at the genus level were either positively or negatively associated with the levels of 20 shared differentially expressed metabolites between the Control vs. Stress and Sham vs. FMT comparisons, including the vitamin B6 metabolites, 4-pyridoxic acid and 4-pyridoxate. Vitamin B6 supplementation during stress alleviated weight loss, abnormal behaviors, peripheral inflammation, and neuroinflammation, but did not affect peripheral corticosterone levels in chronically stressed rats. Dampening inflammatory signaling via knocking out caspase 11 pretreated with caspase 1 inhibitors abolished chronic stress-induced abnormal behaviors in mice. These results showed that gut dysbiosis-induced vitamin B6 metabolism disorder is a novel HPA-independent mechanism underlying chronic stress-related brain disorders. Both probiotics and vitamin B6 supplementation may be potential strategies for preventing and/or treating chronic stress-related illness.

To date, changes in brain function and the gut microbiota in chronically stressed individual have been reported.^7–33^ However, it is still unclear how the gut microbiota induces the development of brain disorders in chronically stressed individuals. In this study, we observed that fecal microbiota transplantation, similar to the experience of stress, markedly affects the profile of plasma metabolites. The differentially expressed metabolites were predominantly associated with KEGG signaling pathways that reflect physiological responses to stress. Spearman correlation analysis revealed a spectrum of positive and negative relationships between shifts in the gut bacterial composition and metabolic changes. This evidence strongly suggests that the gut microbiota plays a regulatory role on plasma metabolites during chronic stress. Among the 20 shared differentially expressed metabolites between the Control vs. Stress and Sham vs. FMT comparisons, two substances involved in vitamin B6 metabolism—4-pyridoxate and 4-pyridoxic acid – were identified. Pathway analysis also revealed that both stress and FMT impacted the vitamin B6 metabolic pathway. The decrease in plasma 4-pyridoxic acid was accompanied by a decrease in vitamin B6 metabolic enzymes within in the intestinal wall. Vitamin B6 supplementation during stress alleviated weight loss, abnormal behaviors, peripheral inflammation, and neuroinflammation, but did not affect peripheral corticosterone levels in chronically stressed rats. Previous studies have shown that vitamin B6 inhibited inflammation^27,34^ and increased the anti-stress effect of magnesium.^26^ Together, these studies suggested that chronic stress-induced gut dysbiosis disturbs vitamin B6 metabolism, subsequently inducing inflammation and the development of brain disorders. Gut dysbiosis-induced vitamin B6 metabolism disorder is a new HPA-independent mechanism of chronic stress-related brain disorders.

Elevated plasma proinflammatory cytokines and glucocorticoids are among the most widely investigated pathogenic activators of chronic stress-induced brain disorders. These two major effectors create a vicious pathogenic cycle.^3,35^ In this study, the deletion of caspase 11 or the inhibition of caspase 1 in mice partly prevented the behavioral abnormalities and upregulation of both peripheral and neuroinflammation induced by chronic stress, but did not alter the increase in corticosterone levels. Moreover, it has been reported that deletion of caspase 1 prevented chronic stress-induced cognitive impairment and depressive-like behaviors, but corticosterone changes were not reported.^36,37^ Caspase 1 and caspase 11 are key molecules modulating canonical and noncanonical inflammatory signaling.^38^ These studies combined with our data reported here suggest that inflammation acts as the downstream effector of corticosterone and plays a key role in the pathogenesis of chronic stress-induced brain disorders.

Chronic stress has a strong effect on the commensal gut microbiota,^6,7,7–9^ and the gut microbiota can modulate brain function via multiple signaling pathways.^39^ Transferring the fecal microbiota from chronically stressed donors to germ-free recipient mice resulted in depression-like behavior in the recipient mice.^15,16^ however, this result in germ-free recipient mice lacked clinical transformation value. And the effect of fecal microbiota transplantation on chronic stress core effectors including proinflammatory cytokines and glucocorticoid were not reported in those studies. In this study, sequential transplantation of rat gut microbiota from rats undergoing various stages of chronic stress to normal rats reproduced chronic stress-induced abnormal behaviors, as well as increased peripheral blood corticosterone and inflammatory cytokines levels. Nurturing a beneficial gut microbiome with probiotics effectively blunted the development of abnormal behaviors and inhibited the increase in peripheral blood corticosterone and inflammatory cytokine levels in chronically stressed rats. Our data suggests a causal relationship between gut dysbiosis and the development of brain disorders in chronically stressed individuals. In addition, we further analyzed the enterotypes of the gut microbiota of rats that underwent chronic stress. At the genus level, there were 20 gut microorganisms with an OTU count higher than 100 that displayed inter-group differences between the normal control group and stressed rats. Among these, the stressed rats exhibited a significantly lower percentage of the top 5 gut microorganisms (g__Lactobacillus, g__Ruminococcus, g__Lachnospiraceae_NK4A136_group, g__norank_f__norank_o__Clostridia_UCG-014, g__Romboutsia) compared to the normal controls. Additionally, a similar percentage change in the top 5 gut microorganisms was observed between rats that underwent four transplantations of stressed cecal contents and the sham-treated controls. However, due to the limitation of the 16S rRNA marker gene sequencing approach, the characteristics of bacterial strains in the enterotypes of chronically stressed rats are unknown and warrant further study.

A previous study documented stress-induced alterations in rat metabolites, with notable examples including kynurenine, monoacylglycerols, and diacylglycerols. However, our study did not observe significant changes in these specific metabolites.^15,40,41^ The possible reason for this discrepancy is unknown. However, we can appreciate that the complexity of microbiota transplantation and conditions in humans all contribute to significant differences on microbiota colonization and transplantation effects.^42,43^ One aspect of the current study that was different from the previous study of FMT via oral gavage is that we used a “clinic” colonoscope approach to avoid gastric acid and oxygen exposure damaging the gut microbiota.^15,41^ The cecum contents were directly delivered via a catheter through the anus to the splenic flexure, pass the hepatic flexure of the colon, and finally into the cecum. Whether the novel transplant mode used in the current study resulted in the different findings is not exclusive and subjected to further study.

Current clinical stress management strategies include distraction, physical activities, relaxation, socializing and healthy food, mindfulness-based stress reduction, and pharmacological approaches.^4^ However, the prevention of brain disorders in chronically stress individuals remains challenging clinically due to poor compliance in nonpharmacologic inventions and side effects of pharmacological approaches. In addition, prevention is further exacerbated due to the ineffectiveness of these strategies on stress-induced HPA axis activation and its downstream innate inflammation and glucocorticoid activation. Here, our study revealed a causal relationship and key role of vitamin B6 between gut dysbiosis and the development of chronic stress-related brain disorders, providing a proof-of-concept for targeting gut dysbiosis with probiotics and inflammation with vitamin B6 supplement to prevent brain disorders in individuals with chronic stress. Both treatments are applicable to patients and further clinical studies to verify their therapeutic value are urgently needed.

Our study has some limitations. First, we found that the levels of plasma vitamin B6 were decreased in chronically stressed rats and FMT rats and were related with some of the gut microbiota at the genus level. Intraperitoneal injection of vitamin B6 in chronically stressed rats effectively prevented gut dysbiosis-plasma vitamin B6 decrease-abnormal behaviors pathway. These results showed the important role gut dysbiosis-induced vitamin B6 metabolic disorder plays in chronic stress-related abnormal behaviors. However, the role of intraperitoneal supplementation of vitamin B6 in FMT animals as compared to oral supplementation was not assessed. In addition, the families Lachnospiraceae, Erysipelotrichaceae, Clostridiaceae, and Corynebacteriaceae, which showed significant abundance changes in previous studies, were not significantly correlated with the vitamin B6 metabolism pathway in this study. No specific bacterial strains were found to be related with vitamin B6. The causal relationship between gut dysbiosis and vitamin B6 metabolic disorder in chronic stress warrants further study.

In conclusion, we demonstrated that chronic stress-induced gut dysbiosis and subsequent systemic and neural inflammation drives the development of brain disorders in chronically stressed individuals and was partially mediated by vitamin B6 depletion. Both probiotics and vitamin B6 supplements can correct such pathological changes and have the potential to serve as strategies to be used clinically.

## Supplementary Material

Supplemental Material

## Data Availability

All of the sequencing data that support the findings of this study have been deposited in the NCBI under accession code PRJNA968156.

## References

[1] Saeedi M, Rashidy-Pour A. Association between chronic stress and Alzheimer’s disease: therapeutic effects of saffron. Biomed Pharmacother. 2021;133:110995. doi:10.1016/j.biopha.2020.110995. 33232931

[2] Decker AM, Kapila YL, Wang HL. The psychobiological links between chronic stress-related diseases, periodontal/peri-implant diseases, and wound healing. Periodontol 2000. 2021;87(1):94–22. doi:10.1111/prd.12381. 34463997 PMC8459609

[3] Rohleder N. Stress and inflammation - the need to address the gap in the transition between acute and chronic stress effects. Psychoneuroendocrino. 2019;105:164–171. doi:10.1016/j.psyneuen.2019.02.021. 30826163

[4] Anghelescu IG, Edwards D, Seifritz E, Kasper S. Stress management and the role of rhodiola rosea: a review. Int J Psychiatry Clin Pract. 2018;22(4):242–252. doi:10.1080/13651501.2017.1417442. 29325481

[5] Agorastos A, Chrousos GP. The neuroendocrinology of stress: the stress-related continuum of chronic disease development. Mol Psychiatry. 2021;27(1):502–513. doi:10.1038/s41380-021-01224-9. 34290370

[6] Bailey MT, Dowd SE, Galley JD, Hufnagle AR, Allen RG, Lyte M. Exposure to a social stressor alters the structure of the intestinal microbiota: implications for stressor-induced immunomodulation. Brain Behav Immun. 2011;25(3):397–407. doi:10.1016/j.bbi.2010.10.023. 21040780 PMC3039072

[7] Gao X, Cao Q, Cheng Y, Zhao D, Wang Z, Yang H, Wu Q, You L, Wang Y, Lin Y, et al. Chronic stress promotes colitis by disturbing the gut microbiota and triggering immune system response. Proc Natl Acad Sci USA. 2018;115(13):E2960–E2969. doi:10.1073/pnas.1720696115. 29531080 PMC5879702

[8] Marin IA, Goertz JE, Ren T, Rich SS, Onengut-Gumuscu S, Farber E, Wu M, Overall CC, Kipnis J, Gaultier A. Microbiota alteration is associated with the development of stress-induced despair behavior. Sci Rep. 2017;7(1):43859. doi:10.1038/srep43859. 28266612 PMC5339726

[9] Xu M, Wang C, Krolick KN, Shi H, Zhu J. Difference in post-stress recovery of the gut microbiome and its altered metabolism after chronic adolescent stress in rats. Sci Rep. 2020;10(1):3950. doi:10.1038/s41598-020-60862-1. 32127581 PMC7054252

[10] Boehme M, van de Wouw M, Bastiaanssen T, Olavarria-Ramirez L, Lyons K, Fouhy F, Golubeva AV, Moloney GM, Minuto C, Sandhu KV, et al. Mid-life microbiota crises: middle age is associated with pervasive neuroimmune alterations that are reversed by targeting the gut microbiome. Mol Psychiatry. 2020;25(10):2567–2583. doi:10.1038/s41380-019-0425-1. 31092898

[11] Yang C, Fujita Y, Ren Q, Ma M, Dong C, Hashimoto K. Bifidobacterium in the gut microbiota confer resilience to chronic social defeat stress in mice. Sci Rep. 2017;7(1):45942. doi:10.1038/srep45942. 28368029 PMC5377462

[12] Burokas A, Arboleya S, Moloney RD, Peterson VL, Murphy K, Clarke G, Stanton C, Dinan TG, Cryan JF. Targeting the microbiota-gut-brain axis: prebiotics have anxiolytic and antidepressant-like effects and reverse the impact of chronic stress in mice. Biol Psychiatry. 2017;82(7):472–487. doi:10.1016/j.biopsych.2016.12.031. 28242013

[13] Westfall S, Caracci F, Zhao D, Wu QL, Frolinger T, Simon J, Pasinetti GM. Microbiota metabolites modulate the T helper 17 to regulatory T cell (Th17/Treg) imbalance promoting resilience to stress-induced anxiety- and depressive-like behaviors. Brain Behav Immun. 2021;91:350–368. doi:10.1016/j.bbi.2020.10.013. 33096252 PMC7986984

[14] De Palma G, Blennerhassett P, Lu J, Deng Y, Park AJ, Green W, Denou E, Silva MA, Santacruz A, Sanz Y, et al. Microbiota and host determinants of behavioural phenotype in maternally separated mice. Nat Commun. 2015;6(1):7735. doi:10.1038/ncomms8735. 26218677

[15] Chevalier G, Siopi E, Guenin-Mace L, Pascal M, Laval T, Rifflet A, Boneca IG, Demangel C, Colsch B, Pruvost A, et al. Effect of gut microbiota on depressive-like behaviors in mice is mediated by the endocannabinoid system. Nat Commun. 2020;11(1):6363. doi:10.1038/s41467-020-19931-2. 33311466 PMC7732982

[16] Li N, Wang Q, Wang Y, Sun A, Lin Y, Jin Y, Li X. Fecal microbiota transplantation from chronic unpredictable mild stress mice donors affects anxiety-like and depression-like behavior in recipient mice via the gut microbiota-inflammation-brain axis. Stress. 2019;22(5):592–602. doi:10.1080/10253890.2019.1617267. 31124390

[17] Liu X, Betzenhauser MJ, Reiken S, Meli AC, Xie W, Chen BX, Arancio O, Marks AR. Role of leaky neuronal ryanodine receptors in stress-induced cognitive dysfunction. Cell. 2012;150(5):1055–1067. doi:10.1016/j.cell.2012.06.052. 22939628 PMC3690518

[18] Wang Z, Chen WH, Li SX, He ZM, Zhu WL, Ji YB, Wang Z, Zhu XM, Yuan K, Bao YP, et al. Gut microbiota modulates the inflammatory response and cognitive impairment induced by sleep deprivation. Mol Psychiatry. 2021;26(11):6277–6292. doi:10.1038/s41380-021-01113-1. 33963281

[19] Cui X, Zhou S, Xia G, Chen J, Jiang L, Huang J, Tong J. A multispecies probiotic accelerates fear extinction and inhibits relapse in mice: role of microglia. Neuropharmacology. 2021;193:108613. doi:10.1016/j.neuropharm.2021.108613. 34022177

[20] Wang P, Yin X, Chen G, Li L, Le Y, Xie Z, Ouyang W, Tong J. Perioperative probiotic treatment decreased the incidence of postoperative cognitive impairment in elderly patients following non-cardiac surgery: a randomised double-blind and placebo-controlled trial. Clin Nutr. 2021;40(1):64–71. doi:10.1016/j.clnu.2020.05.001. 32451125

[21] Qing W, Li F, Wang X, Quan C, Ouyang W, Liao Q. Inhibiting RIP1 improves chronic stress-induced cognitive impairments in D-Galactose-induced aging mice. Front Behav Neurosci. 2018;12:234. doi:10.3389/fnbeh.2018.00234. 30356849 PMC6190884

[22] Li D, Feng Y, Tian M, Ji J, Hu X, Chen F. Gut microbiota-derived inosine from dietary barley leaf supplementation attenuates colitis through PPARγ signaling activation. Microbiome. 2021;9(1):83. doi:10.1186/s40168-021-01028-7. 33820558 PMC8022418

[23] Wei X, Wang L, Hua J, Jin XH, Ji F, Peng K, Zhou B, Yang J, Meng XW. Inhibiting BDNF/TrkB.T1 receptor improves resiniferatoxin-induced postherpetic neuralgia through decreasing ASIC3 signaling in dorsal root ganglia. J Neuroinflammation. 2021;18(1):96. doi:10.1186/s12974-021-02148-5. 33874962 PMC8054387

[24] Casertano M, Dekker M, Valentino V, De Filippis F, Fogliano V, Ercolini D. Gaba-producing lactobacilli boost cognitive reactivity to negative mood without improving cognitive performance: a human double-blind placebo-controlled cross-over study. Brain Behav Immun. 2024;122:256–265. doi:10.1016/j.bbi.2024.08.029. 39163908

[25] Mooney S, Leuendorf JE, Hendrickson C, Hellmann H. Vitamin B6: a long known compound of surprising complexity. Molecules. 2009;14(1):329–351. doi:10.3390/molecules14010329. 19145213 PMC6253932

[26] Pouteau E, Kabir-Ahmadi M, Noah L, Mazur A, Dye L, Hellhammer J, Pickering G, Dubray C, Song Y. Superiority of magnesium and vitamin B6 over magnesium alone on severe stress in healthy adults with low magnesemia: a randomized, single-blind clinical trial. PLOS ONE. 2018;13(12):e208454. doi:10.1371/journal.pone.0208454. PMC6298677 30562392

[27] Shan MR, Zhou SN, Fu CN, Song JW, Wang XQ, Bai WW, Li P, Song P, Zhu ML, Ma ZM, et al. Vitamin B6 inhibits macrophage activation to prevent lipopolysaccharide-induced acute pneumonia in mice. J Cell Mol Med. 2020;24(5):3139–3148. doi:10.1111/jcmm.14983. 31970902 PMC7077594

[28] Walden M, Tian L, Ross RL, Sykora UM, Byrne DP, Hesketh EL, Masandi SK, Cassel J, George R, Ault JR, et al. Metabolic control of BRISC–SHMT2 assembly regulates immune signalling. Nature. 2019;570(7760):194–199. doi:10.1038/s41586-019-1232-1. 31142841 PMC6914362

[29] Ueland PM, Ulvik A, Rios-Avila L, Midttun O, Gregory JF. Direct and functional biomarkers of vitamin B6 status. Annu Rev Nutr. 2015;35(1):33–70. doi:10.1146/annurev-nutr-071714-034330. 25974692 PMC5988249

[30] Shi J, Zhao Y, Wang Y, Gao W, Ding J, Li P, Hu L, Shao F. Inflammatory caspases are innate immune receptors for intracellular LPS. Nature. 2014;514(7521):187–192. doi:10.1038/nature13683. 25119034

[31] McEwen BS. Physiology and neurobiology of stress and adaptation: central role of the brain. Physiol Rev. 2007;87(3):873–904. doi:10.1152/physrev.00041.2006. 17615391

[32] Lupien SJ, Juster RP, Raymond C, Marin MF. The effects of chronic stress on the human brain: from neurotoxicity, to vulnerability, to opportunity. Front Neuroendocrinol. 2018;49:91–105. doi:10.1016/j.yfrne.2018.02.001. 29421159

[33] Dodiya HB, Forsyth CB, Voigt RM, Engen PA, Patel J, Shaikh M, Green SJ, Naqib A, Roy A, Kordower JH, et al. Chronic stress-induced gut dysfunction exacerbates Parkinson’s disease phenotype and pathology in a rotenone-induced mouse model of Parkinson’s disease. Neurobiol Dis. 2020;135:104352. doi:10.1016/j.nbd.2018.12.012. 30579705

[34] Zhang P, Tsuchiya K, Kinoshita T, Kushiyama H, Suidasari S, Hatakeyama M, Imura H, Kato N, Suda T. Vitamin B6 prevents IL-1β protein production by inhibiting NLRP3 inflammasome activation. J Biol Chem. 2016;291(47):24517–24527. doi:10.1074/jbc.M116.743815. 27733681 PMC5114405

[35] Kim YK, Na KS, Myint AM, Leonard BE. The role of pro-inflammatory cytokines in neuroinflammation, neurogenesis and the neuroendocrine system in major depression. Prog Neuropsychopharmacol Biol Psychiatry. 2016;64:277–284. doi:10.1016/j.pnpbp.2015.06.008. 26111720

[36] Towers AE, Oelschlager ML, Lorenz M, Gainey SJ, McCusker RH, Krauklis SA, Freund GG. Handling stress impairs learning through a mechanism involving caspase-1 activation and adenosine signaling. Brain Behav Immun. 2019;80:763–776. doi:10.1016/j.bbi.2019.05.025. 31108171 PMC6664453

[37] Wong ML, Inserra A, Lewis MD, Mastronardi CA, Leong L, Choo J, Kentish S, Xie P, Morrison M, Wesselingh SL, et al. Inflammasome signaling affects anxiety- and depressive-like behavior and gut microbiome composition. Mol Psychiatry. 2016;21(6):797–805. doi:10.1038/mp.2016.46. 27090302 PMC4879188

[38] Van Opdenbosch N, Lamkanfi M. Caspases in cell death, inflammation, and disease. Immunity. 2019;50(6):1352–1364. doi:10.1016/j.immuni.2019.05.020. 31216460 PMC6611727

[39] Fung TC, Olson CA, Hsiao EY. Interactions between the microbiota, immune and nervous systems in health and disease. Nat Neurosci. 2017;20(2):145–155. doi:10.1038/nn.4476. 28092661 PMC6960010

[40] Michels N, Clarke G, Olavarria-Ramirez L, Gomez-Martinez S, Diaz LE, Marcos A, Widhalm K, Carvalho LA. Psychosocial stress and inflammation driving tryptophan breakdown in children and adolescents: a cross-sectional analysis of two cohorts. Psychoneuroendocrinology. 2018;94:104–111. doi:10.1016/j.psyneuen.2018.05.013. 29775873

[41] Jianguo L, Xueyang J, Cui W, Changxin W, Xuemei Q. Altered gut metabolome contributes to depression-like behaviors in rats exposed to chronic unpredictable mild stress. Transl Psychiatry. 2019;9(1):40. doi:10.1038/s41398-019-0391-z. 30696813 PMC6351597

[42] Zmora N, Zilberman-Schapira G, Suez J, Mor U, Dori-Bachash M, Bashiardes S, Kotler E, Zur M, Regev-Lehavi D, Brik RB, et al. Personalized gut mucosal colonization resistance to empiric probiotics is associated with unique host and microbiome features. Cell. 2018;174(6):1388–1405.e21. doi:10.1016/j.cell.2018.08.041. 30193112

[43] Li N, Zuo B, Huang S, Zeng B, Han D, Li T, Liu T, Wu Z, Wei H, Zhao J, et al. Spatial heterogeneity of bacterial colonization across different gut segments following inter-species microbiota transplantation. Microbiome. 2020;8(1):161. doi:10.1186/s40168-020-00917-7. 33208178 PMC7677849

