# Correction

**DOI:** 10.1080/19490976.2025.2518803

**Published:** 2025-06-15

**Article title**: Gut dysbiosis-induced vitamin B6 metabolic disorder contributes to chronic stress-related abnormal behaviors in a cortisol-independent manner

**Authors**: Qing, W., Chen, H., Ma, X., Chen, J., Le, Y., Chen, H., … Tong, J

**Journal**: *Gut Microbes*

**DOI**: https://doi.org/10.1080/19490976.2024.2447824

In the published version of the article, the image for the control group was mistakenly placed in the slot designated for the sham group in Figure 5m. This error has now been corrected, and the updated version has been published. The corrected figure is shown below:

**Figure 5.**
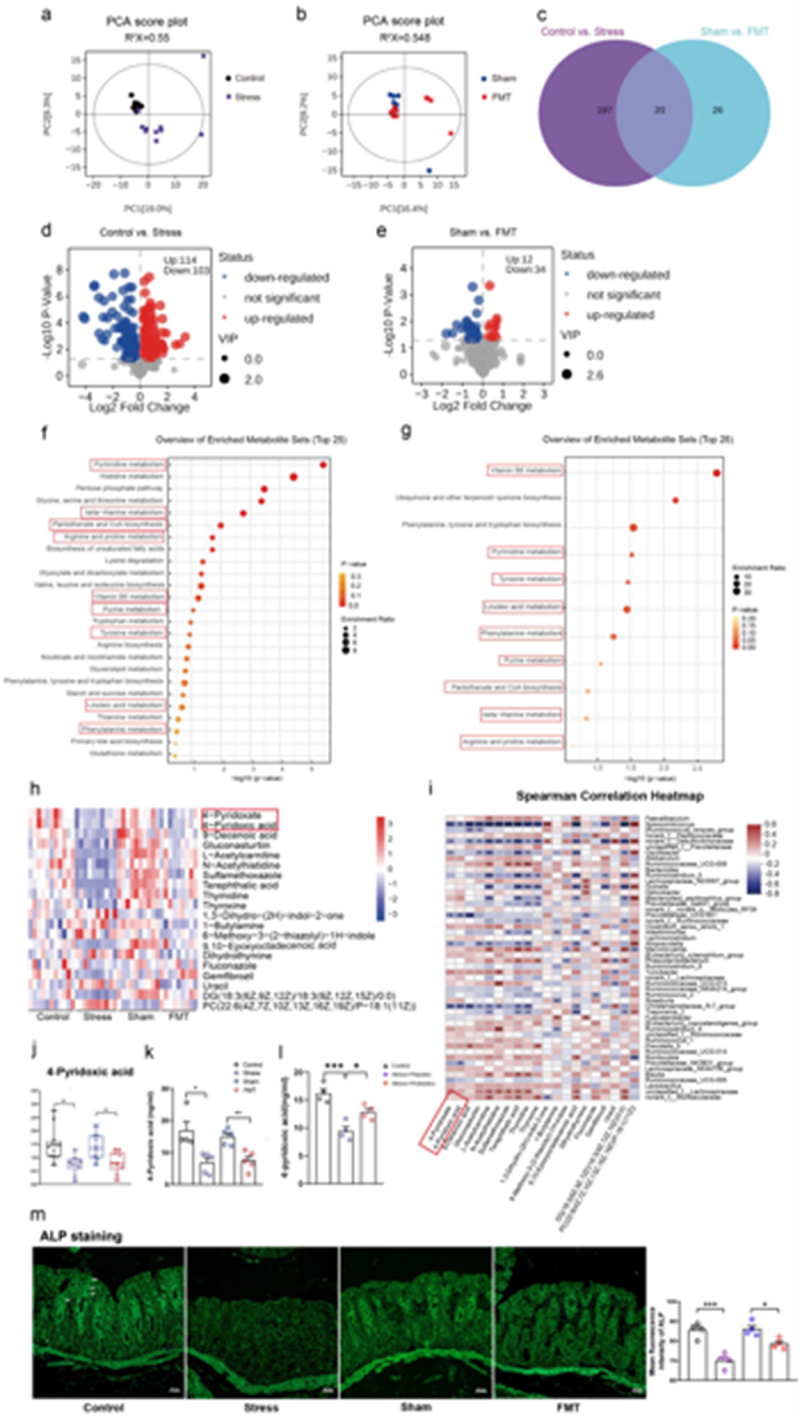
Chronic stress-induced gut dysbiosis altered plasma metabolites, including vitamin B6 metabolism. (a) Principal component analysis (PCA) of the Control vs. Stress groups. (b) Principal component analysis (PCA) of the Sham vs. FMT groups. (c) Venn diagrams reflecting the overlap of various metabolites. (d) Volcano plot analysis of differential metabolites between the Control and Stress groups. (e) Volcano plot analysis of differential metabolites between the Sham and FMT groups. (f) KEGG pathway enrichment analysis of the metabolite profiles of the Control vs. Stress groups. (g) KEGG pathway enrichment analysis of the metabolite profiles of the Sham vs. FMT groups. (h) A heatmap displaying the 20 shared differentially abundant metabolites between the Control vs. Stress and Sham vs. FMT groups. (i) Heatmap of Spearman correlation analysis between the gut microbiota and metabolites. (j) The relative level of 4-pyridoxic acid in the plasma of rats immediately after stress on day 29 in untargeted metabolomics. (k) Targeted validation of the relative level of 4-pyridoxic acid in plasma of the FMT rat group immediately after stress day 29. (l)Targeted validation of the relative level of 4-pyridoxic acid in the plasma of probiotics intervention rat group immediately after stress day 29. (m) Representative micrographs of alkaline phosphatase (ALP) staining in the intestine (white arrow). Scale bar = 50 μm Control: normal control group; Stress: chronic restraint stress group; Sham: sham gut microbiota transplantation group; FMT: fecal microbiota transplantation group.

