# Supplementary material for: Gut dysbiosis-induced vitamin B6 metabolic disorder contributes to chronic stress-related abnormal behaviors in a cortisol-independent manner
Source: Gut Microbes. 2025 Jan 7;17(1):2447824. doi: 10.1080/19490976.2024.2447824 (PMC11730634; doi:10.1080/19490976.2024.2447824)
Supplement: Supplemental Material [file KGMI_A_2447824_SM1020.zip › Supplementary_Materials_.docx]

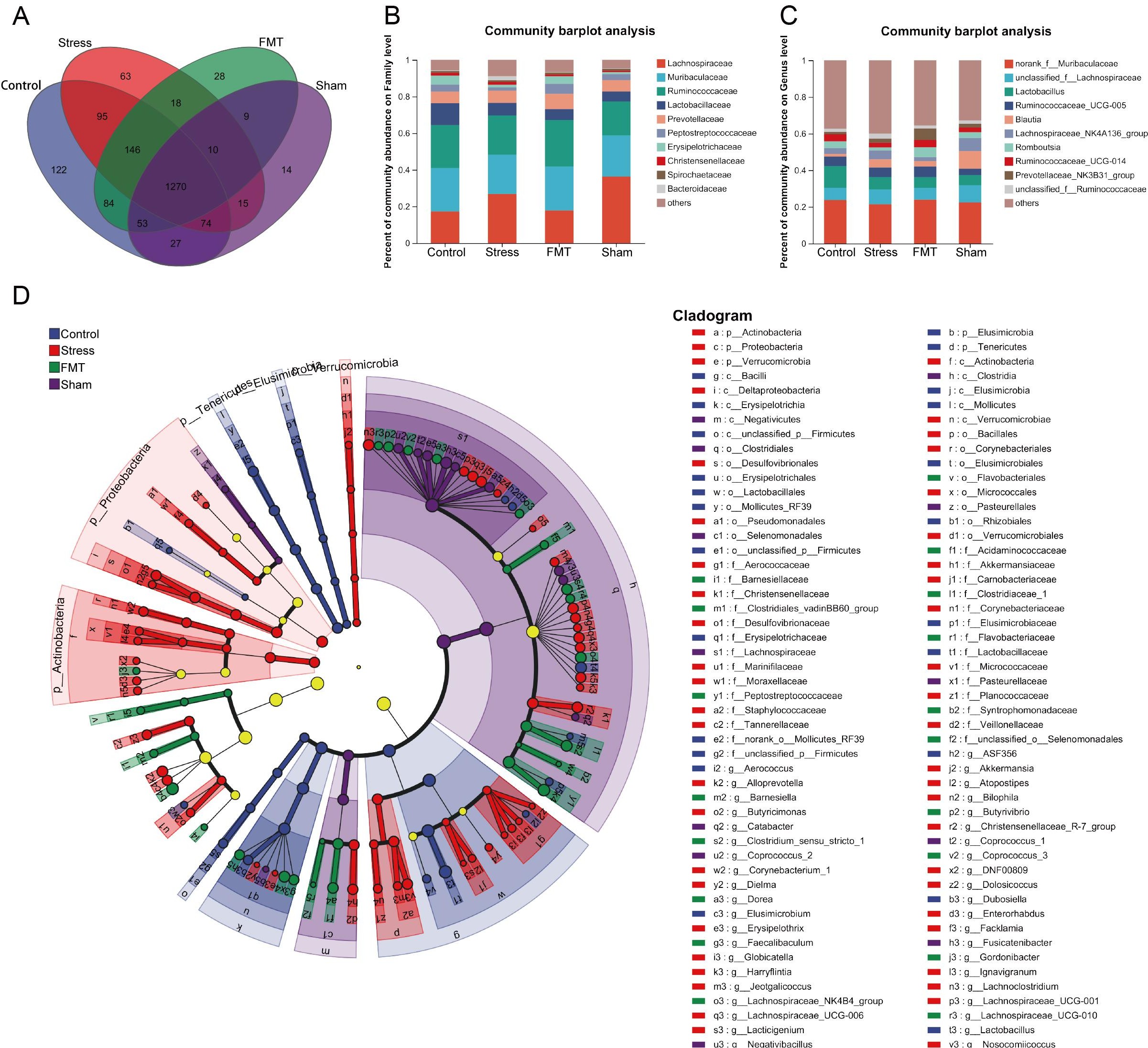


# Figure S1. Sequential transplantation of rat cecal contents of different chronic stress stages to normal rats simulated the gut microbiota dysbiosis typically observed in chronic stress rats.

(A) Venn diagram reflecting the overlap of gut bacteria among different groups. (B) Percentage of family-level community abundance for gut microbiota in the Control, Stress, Sham and FMT groups. (C) Percentage of genus-level community abundance for gut microbiota in the Control, Stress, Sham and FMT groups. (D) LEfSe analysis for gut microbiota in the Control, Stress, Sham and FMT groups.


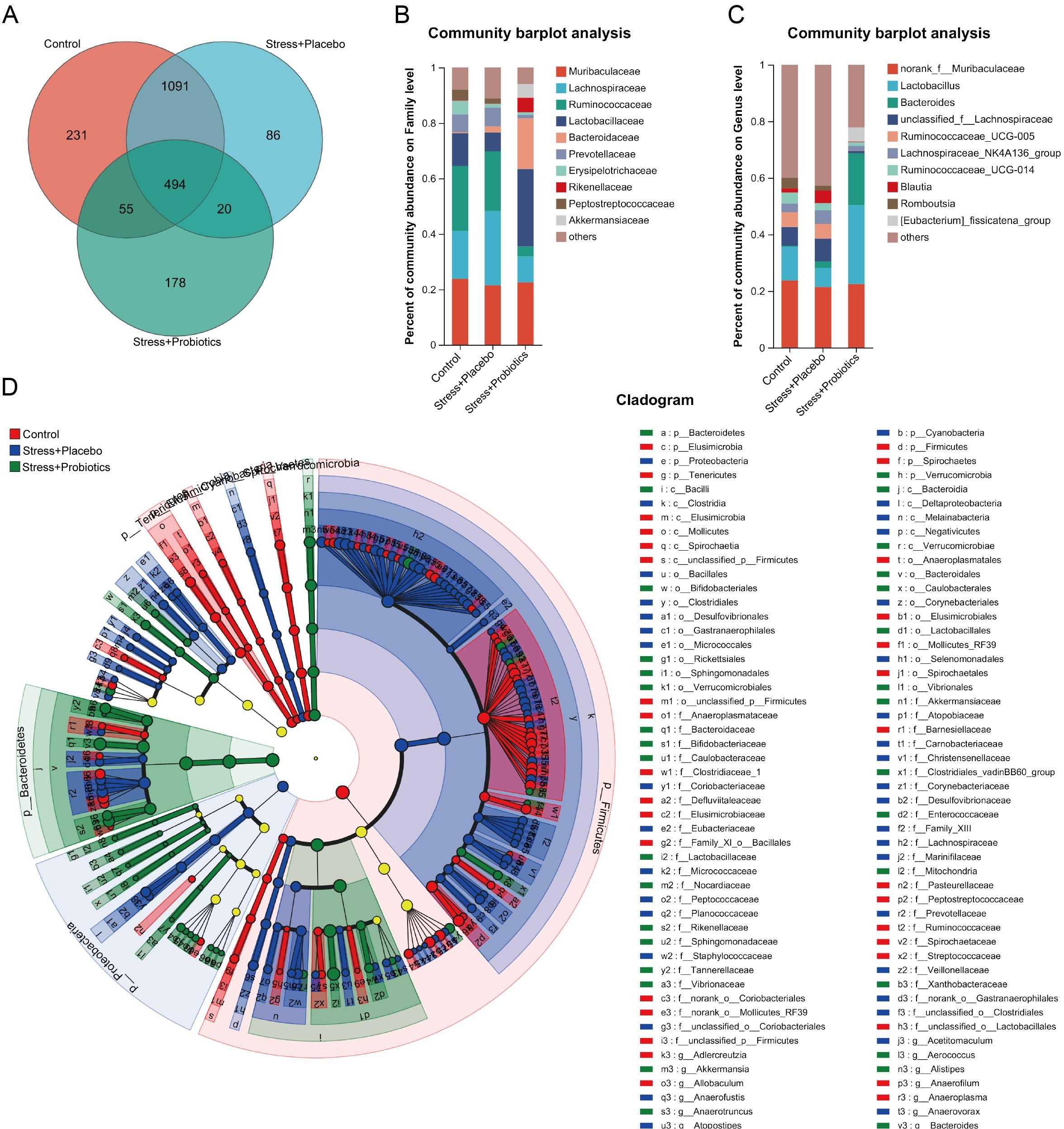


# Figure S2. Probiotics supplementation partially mitigated the intestinal dysbiosis induced by chronic stress.

(A) Venn diagram reflecting the overlap of gut bacteria among different groups. (B) Percentage of family-level community abundance for gut microbiota in the Control, Stress+placebo and Stress+probiotics groups. (C) Percentage of genus-level community abundance for gut microbiota in the Control, Stress+placebo and Stress+probiotics groups.

(D) LEfSe analysis for gut microbiota in the Control, Stress+placebo and Stress+probiotics groups.


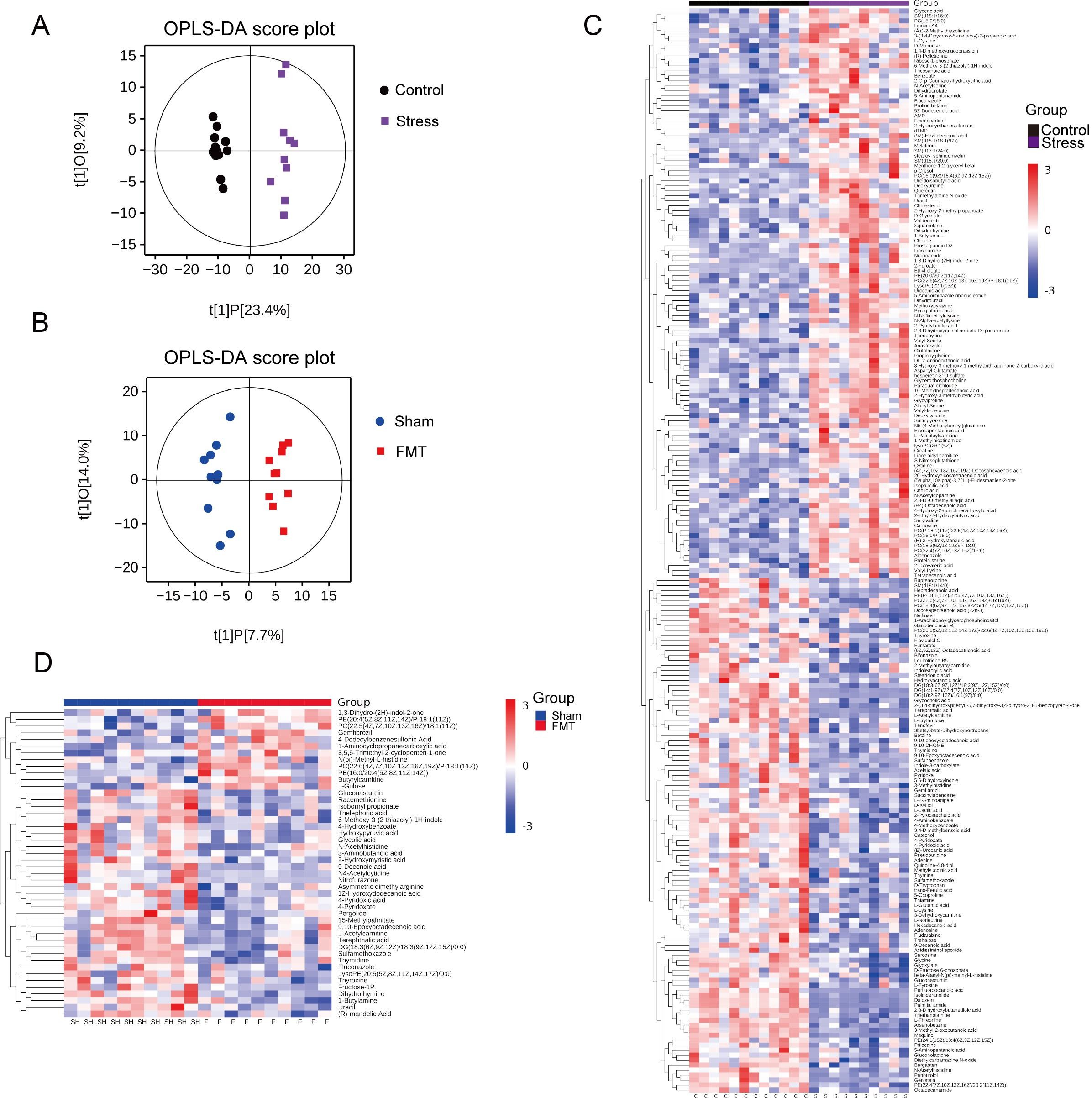


# Figure S3. Stress and FMT each induced significant modifications in the plasma metabolome of rats.

(A) OPLS-DA analysis of the Control and Stress group. (B) OPLS-DA analysis of the Sham and FMT group. (C) Heatmap displaying the distinct plasma metabolites between the Control and Stress groups. (D) Heatmap displaying the distinct plasma metabolites between the Sham and FMT groups.


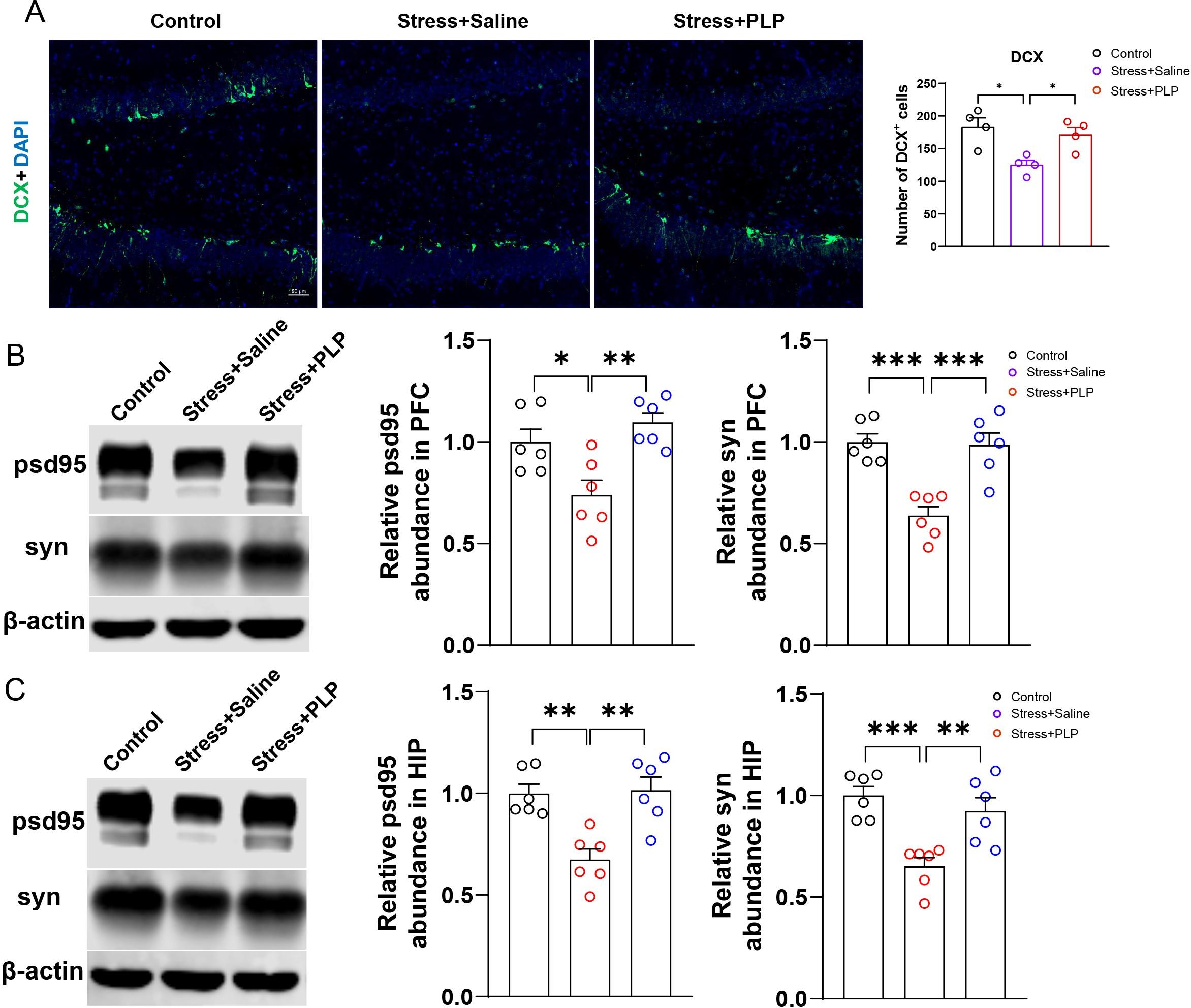


# Figure S4. Vitamin B6 during chronic stress inhibited neuroinflammation and neurogenesis impairment of chronic stressed rats.

(A) Representative images (left panel) and quantification (right panel) of neurogenesis (doublecortin (DCX), green) in hippocampus of rats ( bar=50um). (B) Western blot assays of psd95 and syn in the prefrontal cortex (PFC). (C) Western blot assays of psd95 and syn in the hippocampus (HIP). Data are expressed as mean ± SEM (N=4-6 per group); * p < 0.05, ** p

< 0.01,*** p < 0.005; one- or two-way ANOVA followed by Tukey’s multiple comparison test for comparison or unpaired t test where appropriate. Control: normal control group; Stress+Saline: rats was injected intraperitoneally saline once a day throughout the restraint stress period; Stress+PLP: rats were was injected intraperitoneally PLP once a day throughout the restraint stress period.
